# Flexible data centers reduce power system costs but can increase emissions

**DOI:** 10.1016/j.isci.2026.116497

**Published:** 2026-06-26

**Authors:** Juan Ramon L. Senga, Shen Wang, Christopher R. Knittel

**Affiliations:** 1 Center for Energy and Environmental Policy Research, Massachusetts Institute of Technology, Cambridge MA, USA; 2 MIT Climate Policy Center, Massachusetts Institute of Technology, Cambridge MA, USA; 3 Sloan School of Management, Massachusetts Institute of Technology, Cambridge MA, USA; 4 National Bureau of Economic Research, Cambridge MA, USA

**Keywords:** energy resources, energy policy, energy sustainability

## Abstract

Data centers are among the fastest growing electricity consumers, raising concerns about their impact on grid operations and decarbonization goals. Their temporal flexibility—the ability to shift workloads over time—offers a source of demand-side flexibility. We model power systems in three US regions, Mid-Atlantic, Texas, and Western Interconnect (WECC), under varying flexibility levels. We evaluate flexibility’s effects on grid operations, investment, system costs, and emissions. Across all scenarios, flexible data centers reduced costs by shifting load from peak to off-peak hours, flattening net demand and supporting renewable and baseload resources. This load shifting facilitates renewable integration while improving the utilization of existing baseload capacity. As a result, the emissions’ impact depends on which effect dominates. Higher renewable penetration increases the emission reduction potential of data center flexibility, while lower shares favor baseload generation and may raise emissions. Our findings highlight the importance of aligning data center flexibility with renewable deployment and regional conditions.

## Highlights

• Data center temporal flexibility is modeled for Texas, Mid-Atlantic, and WECC
• Data center temporal flexibility shifts load such that net load is flattened
• Temporal flexibility reduces costs but emissions’ impact is dependent on renewables
• Temporal flexibility can also benefit coal plants by reducing ramping operations

## Graphical abstract

**Figure:**
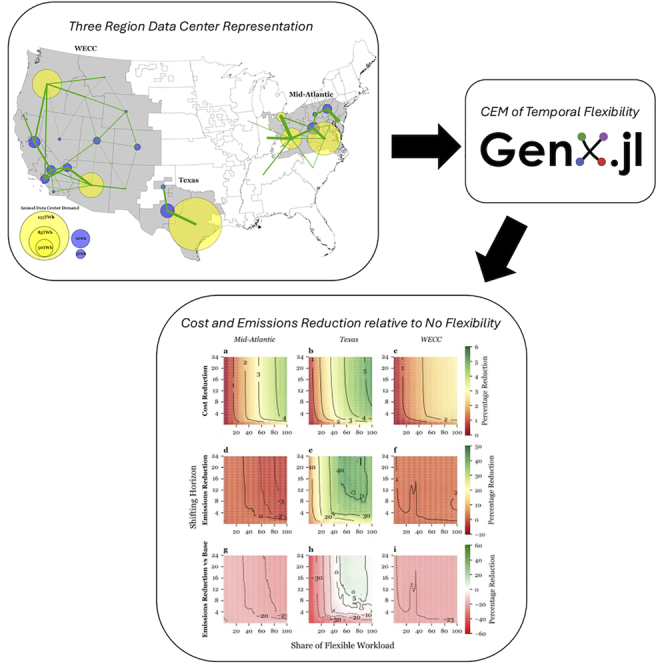

## Introduction

Data centers are among the fastest growing electricity consumers, with their energy demand projected to increase over the coming years.^1^^,^^2^ In the US, that projection is an increase of 7%–12% by 2030.^3^ This surge is driven by advances in artificial intelligence (AI) and the prevalence of cloud computing, which poses challenges for grid reliability^4^ and decarbonization efforts.^5^ The additional load could put stress on the grid and increase the usage of existing thermal power plants, which may increase carbon emissions. For example, in PJM (Pennsylvania-New Jersey-Maryland Regional Transmission Organization), the forecasted increase of 32 GW (20% increase) in summer peak load mostly comes from data centers and is equivalent to adding another mid-sized state’s demand to the system.^6^ However, opportunities exist to operate data centers more flexibly as demand response resources, potentially mitigating large load impacts. One of these strategies takes advantage of a latent demand response resource that we call “data center temporal flexibility”—the ability of data centers to change its load profile by shifting workload across time.^7^ Data centers do not operate at full capacity all the time and typically maintain utilization rates of around 80%.^8^ This is especially true for AI training, which has a relatively flat workload pattern. This gives a headroom of 20% of data center capacity to accommodate additional shifted data center load. Operationally, there could be benefits to shift tasks to hours when renewable availability is high or prices are low.^9^^,^^10^ This may not only save operating costs for data centers but also provide flexibility and increase reliability for the power system while also meeting climate goals.^11^^,^^12^

Prior work hints at these benefits—curtailment relief, renewable firming, and even 24/7 carbon-free alignment when spatial shifting across data center networks is layered on.^5^^,^^13^^,^^14^^,^^15^^,^^16^^,^^17^^,^^18^ Google’s carbon-aware scheduler offers a high-profile proof-of-concept,^10^ and market-based signals appear decisive in whether load-shifting cuts or raises emissions.^19^ Strikingly, even modest flexibility could offset most of the projected US data center growth without a single new power plant.^20^ There is also increasing interest in flexibility as a means to reduce interconnection barriers. NVIDIA recently announced a partnership with Emerald AI to develop software that schedules data center computation, while Google has engaged in demand response initiatives with utilities to alleviate grid strain.^21^^,^^22^ These efforts reflect a broader strategic alignment between flexibility-oriented operations and improved interconnection prospects.

But, several critical research questions remain unanswered. First, it is unclear how data center flexibility affects power system planning and operations. The ability to shift demand could significantly impact investment decisions, plant retirements, and operational strategies. This may alter the trajectory of capacity expansion and reliability planning for regional operators. Second, the potential grid benefits that flexible data centers bring are not yet understood for different levels of flexibility. While some portions of the data center load are flexible, the degree to which it can be shifted is constrained over time. Tasks cannot be postponed indefinitely, and certain tasks may not be shifted at all. Thus, understanding the combinations of flexibility levels (in terms of duration and shifting potential) that can lower cost and emissions is critical. Furthermore, the impact of data center flexibility on different regions may vary depending on the characteristics of the regional grid.

To address these questions, we use the GenX capacity expansion model (CEM). GenX is the least cost model that co-optimizes generation investment, retirement, and operational decisions in the power system for a representative year of operation.^23^ It has been used extensively to assess policy and technology impacts on the grid (see Manocha et al., 2025; Senga et al., 2025; Botterud et al. 2024; Sepulveda et al. 2018^24^^,^^25^^,^^26^^,^^27^^,^^28^ for example). We modified the GenX code base to accommodate different data center temporal flexibility scenarios (see STAR Methods). We model combinations of scenarios by varying the “shifting horizon”—the time window in which loads can be shifted—from 1 to 24 h, and the “share of flexible workload”—the fraction of total shiftable demand (20% of total gross demand)—from 1% to 100%. The final GenX model then includes decisions on the hourly shifting of flexible data center load, limited by the level of flexibility in each scenario. We also include a baseline case without flexibility for comparison and assume that without flexibility, data centers have constant load throughout the year.

**Figure 1:**
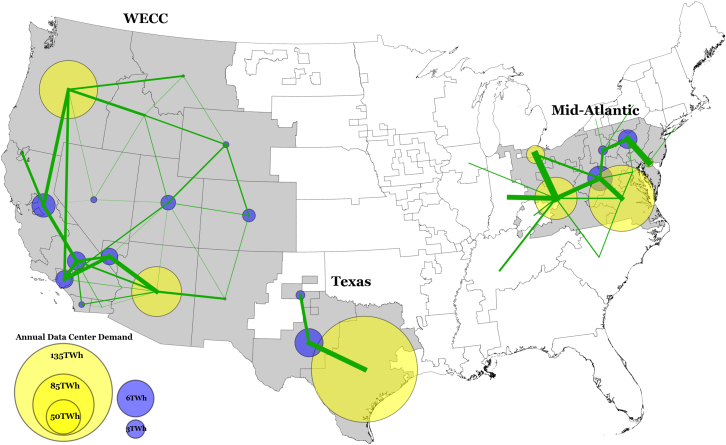
Model zones with transmission lines and data center load Yellow and blue circles represent annual data center demand per zone. Green line segments indicate the transmission links between zones in a region where the width is scaled to the capacity of the line. Detailed information on load assumptions can be found in Note S5.1.

Our testbeds—Texas, the Mid-Atlantic, and the Western Interconnect (WECC)—collectively host 82% of the nation’s projected 2030 data center demand.^5^^,^^17^ Figure 1 visualizes zonal loads and transmission corridors.

Our findings show that data center temporal flexibility can significantly change a power system’s operations and generation mix. Higher flexibility levels enable net load shifting from peak to off-peak hours, flattening the net load profile (net load is defined as the demand net of variable renewable energy [VRE] generation and curtailment). This reduces reliance on peaker or ramping plants and promotes more stable operation of base load generators. When renewables are sufficiently cost competitive—as in Texas where wind and solar are projected to supply 54% of generation—high levels of data center flexibility result in up to 40% lower CO_2_ emissions and accelerate retirements of coal and nuclear plants. This reverses in the Mid-Atlantic and WECC where renewable penetration is lower, coal units that survive retirements can run more uniformly, and system-wide emissions rise by as much as 3%, even though costs still fall.

We confirm this cost sensitivity in a counterfactual experiment that raises renewable investment and fixed operating and maintenance (O&M) costs in Texas to 1.3 times baseline values. Renewable share collapses to 21%, coal plants remain on the system, and the emission advantage of flexibility disappears, demonstrating that data center load shifting substitutes for baseload when clean energy is economical.

Across all regions and price scenarios, however, temporal flexibility “always” lowers the total system costs—by up to 5% in Texas—while steering new investment toward renewables (wind in Texas and solar in WECC and the Mid-Atlantic) and crowding out battery storage. Flexible data center operations, thus, emerge as a robust, low-cost reliability resource whose climate value hinges on the underlying economics of clean power.

To our knowledge, this is the first study to trace “end-to-end” consequences—from grid build-out to hourly dispatch—of data center flexibility. The results show that it can either accelerate decarbonization or entrench fossil fuels that it seeks to displace.

## Results

### The impact of data center flexibility on data center and system operations

#### Data center and grid operations

**Figure 2:**
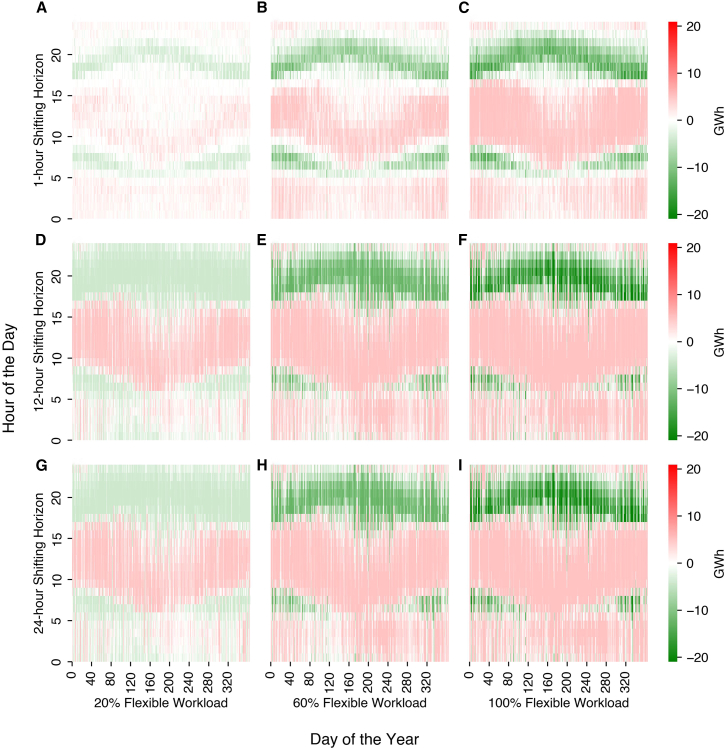
Hourly data center load change in the Mid-Atlantic (A–I) Shown in the hourly change in data center electricity demand (GWh) relative to the baseline across all 8,760 h of the year. *x* axis denotes the day of the year (1–365), and *y* axis denotes the hour of the day (0–23). Red cells indicate hours in which load is added (workload shifted in), and green cells indicate hours in which load is reduced (workload shifted out).Columns correspond to the share of total data center load eligible for shifting: 20% (left), 60% (center), and 100% (right). Rows correspond to the maximum shifting horizon. The farthest ahead or behind a computational task may be rescheduled: 1 h (top), 12 h (middle), and 24 h (bottom).

**Figure 3:**
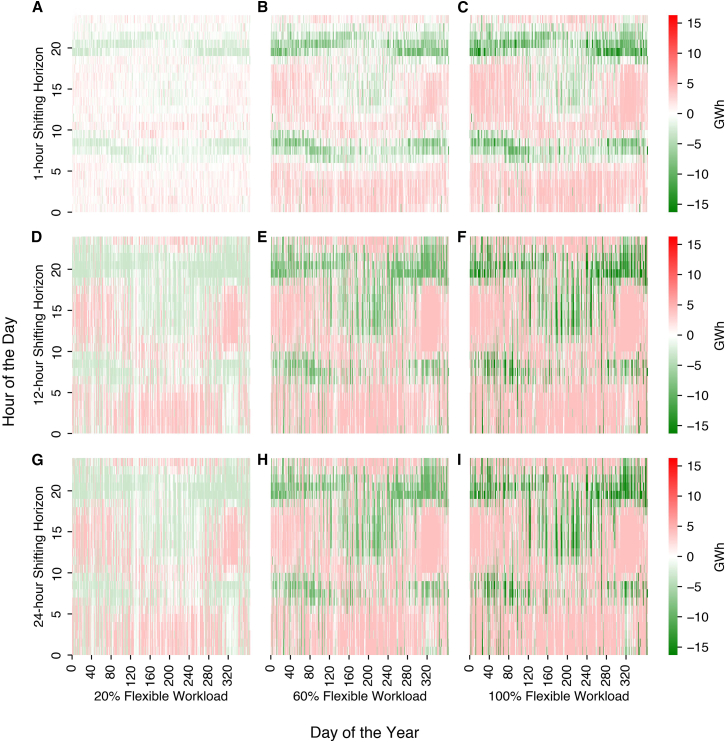
Hourly data center load change in Texas (A–I) Shown is the hourly change in data center electricity demand (GWh) relative to the baseline across all 8,760 h of the year. *x* axis denotes the day of the year (1–365), and *y* axis denotes the hour of the day (0–23). Red cells indicate hours in which load is added (workload shifted in). Green cells indicate hours in which load is reduced (workload shifted out). Columns correspond to the share of total data center load eligible for shifting: 20% (left), 60% (center), and 100% (right). Rows correspond to the maximum shifting horizon. The farthest ahead or behind a computational task may be rescheduled: 1 h (top), 12 h (middle), and 24 h (bottom).

**Figure 4:**
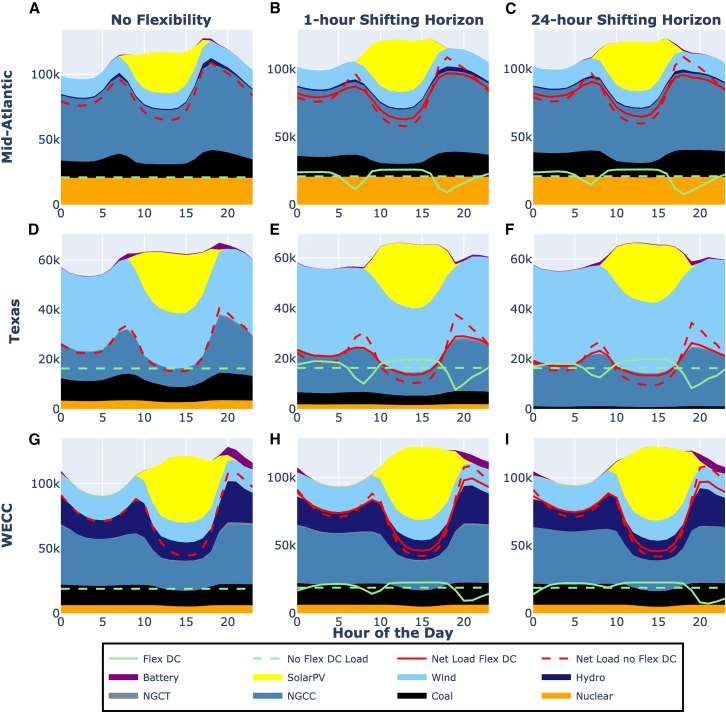
Average winter operations per region (A–I) Each subplot shows the average generation per technology, net load, and data center load shifts in MWh per hour of the day during the winter season. Images correspond to three levels of operational flexibility: no flexibility (A, D, and G), 1-h shifting horizon (B, E, and H), and 24-h shifting horizon (C, F, and I) with a 100% share of flexible workload. The graphs do not include the regions’ net electricity imports. This is relevant for the Mid-Atlantic where we assume deterministic hourly net imports. Details on net import data can be found in Note S5.4.

**Figure 5:**
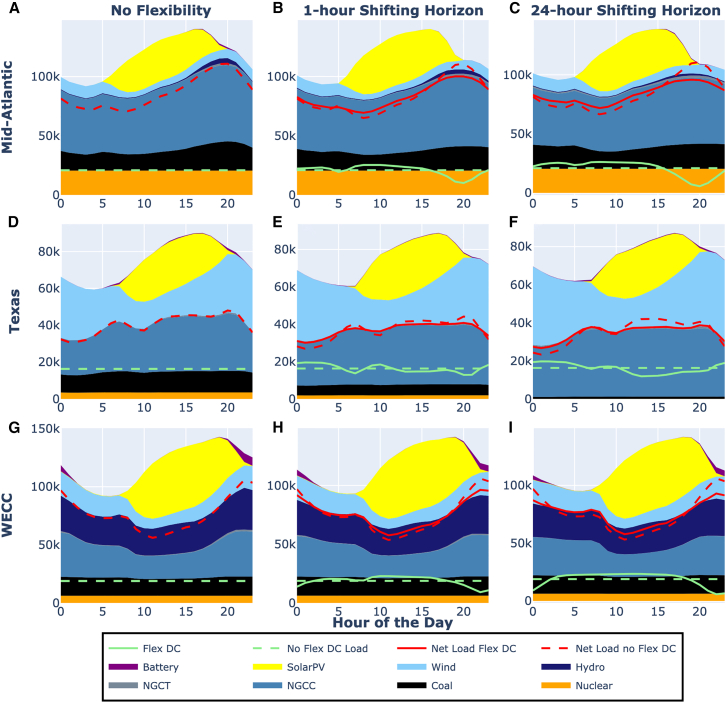
Average summer operations per region (A–I) Each subplot shows the average generation per technology, net load, and data center load shifts in MWh per hour of the day during the summer season. Images correspond to three levels of operational flexibility: no flexibility (A, D, and G), 1-h shifting horizon (B, E, and H), and 24-h shifting horizon (C, F, and I) with a 100% share of flexible workload. The graphs do not include the regions’ net electricity imports. This is relevant for the Mid-Atlantic where we assume deterministic hourly net imports. Details on net import data can be found in Note S5.4.

We first look at how data centers shift their load given different combinations of temporal flexibility. Figures 2 and 3 show the entire year’s data center load shifting operations for the Mid-Atlantic and Texas, respectively, while Figure S5 shows those for WECC. We also show in Figures 4 and 5 the interaction of data center load shifting and power system dispatch across the three regions for average summer and winter conditions, respectively.

We generally observe that the data center load is temporally shifted out of daily peak load hours so that it can flatten net load. For example, in all regions, we find consistent patterns of shifting from early morning hours and early night hours to midday during the winter (Figures 2 and 3; Figure S5). This aligns with reducing the two peaks during these periods and shifting demand to midday hours with high solar availability (Figure 4). Notably, the presence of storage and hydro (purple and dark blue, respectively, in Figure 4I) in WECC further complements flexible load by reducing the net load volatility. In all cases, flexible data center load reduces system ramping requirements. We see this from the flattening of the net load curve when comparing the dashed and solid red lines in Figure 4.

Comparing the “no flexibility” subplots in Figure 5 (Figures 5A, 5D, and 5G) with those incorporating 1- and 24-h shifting horizons (middle Figures 5B, 5E, and 5H, and right Figures 5C, 5F, and 5I columns), we observe a clear flattening of the net load curve (red lines) as flexibility increases. This operational shift leads to notable changes in resource utilization: peaking gas units (NGCT) are dispatched less frequently, while baseload and mid-merit units—such as NGCC, coal, and nuclear—operate more uniformly. The alignment of data center load with solar generation also increases the use of solar. Battery dispatch also becomes less prominent as flexible load partially substitutes its role in balancing variability. Across regions, Texas notably differs from the Mid-Atlantic and WECC. While workloads are shifted from nighttime to midday during the summer for the Mid-Atlantic and WECC (Figure 2; Figures 5C and 5I; Figure S5), data center load is frequently shifted away from the midday in Texas (Figures 3 and 5E). This difference is driven by Texas’ midday net load peaks (dashed red line, Figure 5D), which is unique in the three regions as Texas has high cooling demand during these hours and limited baseload generation (Figures 5E and 5F). In contrast, more baseload nuclear and coal capacity in the Mid-Atlantic and WECC leads to net load peaks that are later in the day. Flexible data center loads then tend to be shifted into midday hours, leveraging lower marginal costs from solar generation and avoiding evening ramp pressures (Figures 5C and 5I). We also observe a reduction in coal and nuclear generation in Texas with more flexibility, leading up to almost no baseload generation at a 24-h shifting horizon (Figures 5D, 5E, and 5F). Regional system characteristics, such as resource mix and load shape, thus influence optimal data center shifting operations.

Finally, we note that the load shifting is more localized in the 1-h shifting horizon case as load is constrained to a smaller time window but becomes more pronounced with a 12- or 24-h shifting horizon (for example, see Figures 2A, 2D, and 2G for the Mid-Atlantic). This also implies that the 24-h and 100% share of flexible workload scenario (Figure 2I) shows the most extensive redistribution of data center load. However, even modest flexibility (12-h shifting horizon and 60% share of flexible workload; Figure 2E) in data center operations can already lead to significant grid re-balancing.

#### Capacity and generation mix

**Figure 6:**
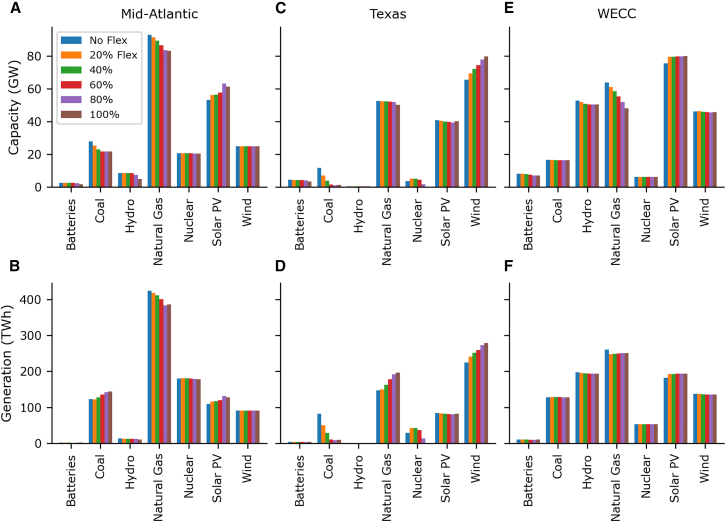
Capacity and generation per region with 24-h shifting horizon (A, C, and E) Total installed capacity by technology, accounting for new investments and retirements. (B, D, and F) Corresponding total generation by technology. Results are shown across increasing flexible workload shares (20%–100% in 20% increments), assuming a 24-h shifting horizon, alongside a baseline scenario without flexibility. Coal, nuclear, and hydro are excluded from new capacity additions but remain eligible for retirement. Data on capacity retirements and investments at all flexibility combinations can be found in Notes S6 and S7.

Data center flexibility and the redistribution of net load naturally affect the capacity and generation mix of a power system. Figure 6 shows the capacity and generation per technology at varying levels of data center flexibility for each region.

Overall, we observe two main effects. First, flexibility supports renewable investments. By shifting demand into hours with high renewable availability, data center flexibility increases the economic value of wind and solar generation. This leads to larger solar investment in the Mid-Atlantic (and to a lesser extent in WECC) and larger wind investment in Texas. The preference for either is driven by which resources are more abundant in the region. Texas has strong wind potential, while the Mid-Atlantic and WECC are better suited to solar. Second, data center flexibility supports baseload operations. By flattening net load profiles, data center flexibility makes it more cost effective to run inflexible baseload plants like coal with fewer ramping requirements.

Whether the natural gas capacity and generation increase or decrease depends on which of these two effects dominates. In the Mid-Atlantic and WECC, the support for baseload is stronger. This reduces the need for flexible natural gas capacity as coal generation becomes more economically viable. In contrast, in Texas, the support for renewables dominates due to the high share of renewable generation of around 54% (39% wind and 15% solar) of total mix, compared with 22% (10% wind and 12% solar) in the Mid-Atlantic and 33% (14% wind and 19% solar) in WECC. This drives an increase in natural gas generation that serves as a flexible, fast-ramping complement to wind. As a result, Texas sees less reliance on baseload plants like coal and nuclear. This is reflected in coal and nuclear retirements and reduced generation. Interestingly, in the Mid-Atlantic, coal retirements also increase, but the baseload support of data center flexibility leads to higher coal generation.

#### The impact of data center flexibility on carbon emissions

The results of our modeling show that the projected 2030 data center load growth relative to a system with no data center growth increases annual CO_2_ emissions by 47 Mmt (20%), 55 Mmt (58%), and 46 Mmt (24%) for the Mid-Atlantic, Texas, and WECC regions, respectively. The increase in projected emissions emphasizes the urgency of identifying strategies to reduce data centers’ environmental impact, particularly in evaluating if data center flexibility can lead to emission mitigation.

However, the environmental consequences of the shifts in generation and capacity induced by data center flexibility are not straightforward. As shown in the previous section, flexibility can simultaneously promote both renewable deployment and greater utilization of inflexible baseload generators. This dual effect raises a natural question: does data center flexibility reliably reduce emissions, or can it, under certain conditions, lead to the opposite? While the prevailing view is that any form of demand flexibility complements the use of clean energy, our results suggest this intuition may not always hold. In this section, we examine how emission outcomes depend on the interplay between flexibility and the underlying generation mix. We find that temporal flexibility in data centers does not always reduce the total annual CO_2_ emissions relative to a system without flexibility.

**Figure 7:**
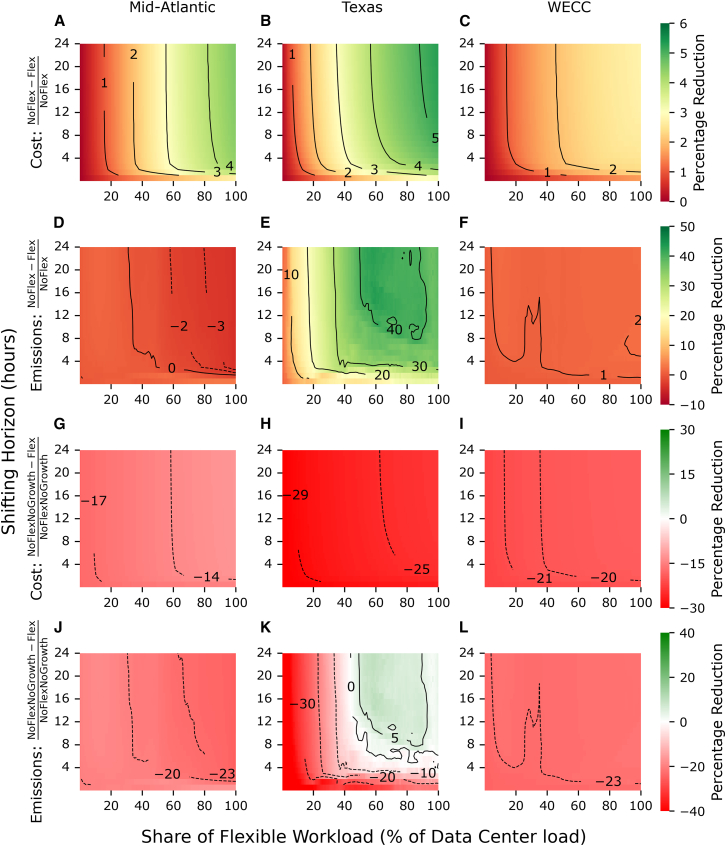
Cost and emission reductions (A–C) Heatmaps showing the percentage reduction in total system cost from introducing flexible data centers, relative to a system without flexibility, across combinations of shifting horizon and share of flexible workload. (D–F) Heatmaps of percentage reduction in system CO_2_ emissions under the same flexibility configurations, relative to the no flexibility baseline. (G–I) Heatmaps of percentage reduction in total system relative to a reference system with no data center flexibility and no data center load growth. (J–L) Heatmaps of percentage reduction of system CO_2_ emissions relative to a reference system with no data center flexibility and no data center load growth. Green (red) color indicates a decrease (increase) in CO_2_ emissions relative to the no growth scenario. The “no growth” baseline assumes data center load in 2030 maintains the same share of total system load as in 2022. Details on load assumptions are found in Note S5.1. Color scales vary across rows.

Figures 7D. 7E, and 7F show the percentage reduction in emissions of systems with flexible data centers compared with those in systems without that flexibility. In Texas, emissions fall significantly by up to 40%. But in the Mid-Atlantic, we observe a counterintuitive result: greater data center flexibility leads to higher CO_2_ emissions. In systems with high renewable penetration and limited remaining coal capacity, flexibility mostly enables greater renewable utilization and emission reductions (i.e., Texas). Data center flexibility can have a significant impact on emissions when coupled with high renewable penetration, such that even with projected growth in data center load, a flexible system can achieve lower emissions than a system without either data center growth or flexibility (Figure 7K). In contrast, in systems with a large share of existing coal and relatively limited VRE availability, flexibility tends to shift load toward cheap, carbon-intensive baseload generation, which raises emissions even as costs fall (Figure 7D).

**Figure 8:**
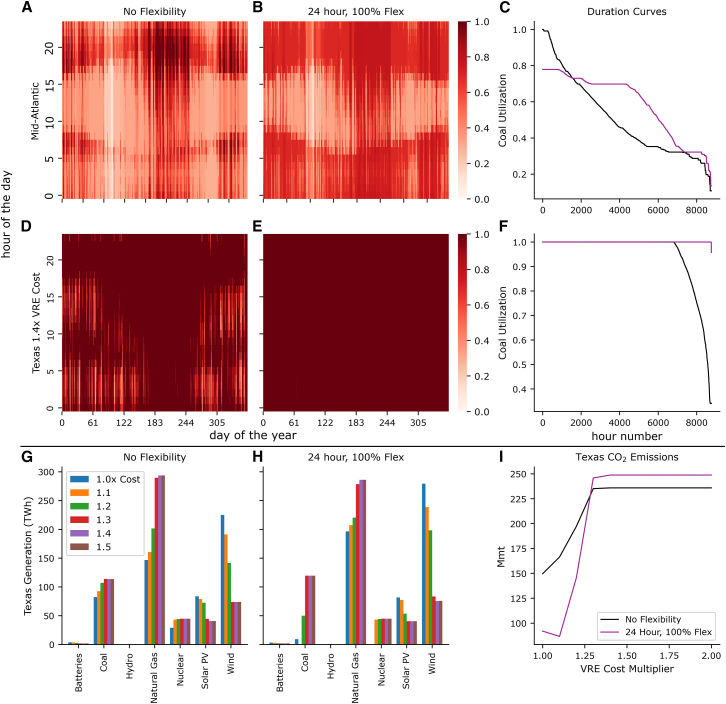
Coal utilization and emissions under varying VRE costs and data center flexibility (A and B) Heatmaps of coal utilization in the Mid-Atlantic under no flexibility and with a 24-h shifting horizon with 100% flexible workload. (C–F) Duration curves ranking hourly coal output in descending order across all 8,760 h. (D) and (E) show analogous results for Texas under a 40% increase in VRE costs. Coal utilization is normalized using a common capacity baseline—the higher of the two scenarios’ remaining coal capacity—to ensure consistent comparison. (G and H) Average generation by technology in Texas under varying VRE cost multipliers, which affect investment and fixed O&M costs for new wind and solar. (I) Total system CO_2_ emissions across the same cost multipliers. The base model is represented by a cost multiplier of 1.0.

This trade-off is evident in the Mid-Atlantic. Initially, with flexibility, load shifts to hours with high VRE generation, which reduces thermal dispatch and variable O&M costs—the first effect. However, once VRE potential is exhausted, the second effect emerges, with additional load shifted to hours when cheap thermal generation is available, particularly baseload coal (Figures 5A and 5C). With full flexibility, average hourly coal utilization in the Mid-Atlantic rises from 50% to 59%. Figures 8A and 8B show heatmaps of hourly coal utilization in the Mid-Atlantic. Without flexibility, coal ramps up in the evening and ramps down during the day when solar generation is high (Figure 8A). The summer evenings see the largest utilization of coal as this time period coincides with the highest net loads. With flexibility, coal’s output gets distributed more evenly from the evening to the early morning (Figure 8B). The high summer evening utilization of coal is lowered, and utilization throughout the rest of the day is increased as evening data center load gets shifted to the morning (Figure 2). A duration curve reveals that coal operates between 68% and 78% utilization for 4,526 h (52% of the year) with flexibility, compared to just 898 h (10%) without flexibility (Figure 8C). This shift to steadier coal operations contributes to higher total emissions.

To further illustrate that the emission impact is driven by the availability of renewables, we simulate an alternative set of Texas scenarios where the investment and fixed O&M costs of renewables are increased. In these systems with less economically viable renewables, we expect and find that the share of renewables decreases. More importantly, we see that the emission-reducing effect of flexibility is reversed. Data center flexibility increases emissions relative to an inflexible system by up to 5% at renewables that cost 1.3 times the base prices (Figure 8I). In this system, as in the Mid-Atlantic, insufficient renewable capacity to absorb flexible demand results in coal not being retired and instead becoming more heavily utilized with less ramping (Figures 8D and 8E).

These findings show that the generation mix determines whether data center flexibility reduces or increases emissions. This also parallels the results on battery storage where it can produce counterintuitive emission outcomes under certain conditions.^29^ In systems with high renewable penetration and potential like Texas, flexibility typically aligns with wind and solar, displacing thermal generation and lowering emissions. As a consequence, the lower emission outcome is not guaranteed and is contingent on the availability and build out of renewable resources. The emission impact of data center flexibility is, thus, not inherent to flexibility itself, but rather depends on the surrounding resource mix and investment environment. Flexibility consistently reduces system costs, but without adequate clean generation, it can inadvertently increase system emissions by reinforcing baseload coal operations.

#### The impact of data center flexibility on cost

A key output of the GenX model is the optimal total annual system cost. This consists of investment, fixed and variable O&M costs, fuel, startup costs, and applicable tax credits. Figures 7A, 7B, and 7C are heatmaps of the percentage reduction in total system cost of a system with flexible data centers relative to the “no flexibility” scenario for the Mid-Atlantic, Texas, and WECC regions, respectively. Meanwhile, Figures 7G, 7H, and 7I show the same comparison but relative to a system with neither data center flexibility nor load growth.

We first highlight that the increase in data center load leads to an increase in the overall system cost. The increase can be substantial, by up to 30% in Texas. However, data center temporal flexibility can alleviate a portion of this increase. That is, data center temporal flexibility leads to lower total system costs relative to systems without flexibility. Increasing the shifting horizon and the share of flexible workload also increases the cost savings, with reductions of up to 4% in the Mid-Atlantic, 5% in Texas, and 2% in WECC compared with a no flexibility scenario. The cost reduction is primarily constrained by the share of flexible workload, rather than the shifting horizon. The contour lines indicate that achieving specific cost reduction levels is only possible over certain ranges of the share of flexible workload. For example, in Texas, a 2% reduction in costs cannot be achieved if only 10% of data center load can be shifted to other hours, no matter how long is the shifting horizon. To achieve that 2% reduction, the share of flexible workload has to be increased to a value between 21% and 49%. The relationship between flexibility and cost reduction is also non-linear, with an additional share of flexible workload yielding diminishing returns.

While data center flexibility reduces cost at all levels of flexibility, the source of these savings varies per region. Figures S7, S8, and S9 show the cost difference per cost component relative to a “no flexibility” scenario for the Mid-Atlantic, Texas, and WECC, respectively. In the Mid-Atlantic, the savings come from a reduction in investments in new natural gas (Figures 6A and S2D) and the retirement of a portion of the existing coal plants (Figures 6A and S1B). With the lower investment in new natural gas capacity, the system avoids a generation mix that would need to be spend on fuel costs that it would otherwise incur in an inflexible system. These lower investments and the additional retirements of coal generation also avoid fixed O&M costs. Note, however, that the increase in operation of the remaining non-retired coal plants with additional flexibility (Figure S6B) increases the variable O&M costs.

In Texas, the cost benefits of temporal flexibility rely on aligning data center load with cheap VRE resources. When there is a larger share of load that can be shifted to hours with high VRE availability factors, it results in lower spending on fuel and variable O&M for thermal generators. However, this increased share of renewables is possible only by building new wind capacity, which increases the investment cost (Figures 6C and S3B). At relatively small shares of flexible workload (i.e., ≤50%), Texas systems with flexibility increase fixed O&M cost because of the non-retirement of nuclear plants (Figures 6C andS1D). Similar insights to those in Texas can be found in WECC. The only difference is that the increase in investments primarily comes from solar rather than wind, which slightly goes down as flexibility increases. This reduction in wind investments (Figures 6E andS4B) and an increase in natural gas retirements (Figures 6E andS1I) with more flexibility is what drives the lower fixed O&M costs.

#### Sensitivity analysis

Our results show that the relative cost efficiency between VRE technologies and baseload plants is the key in determining whether data center flexibility results in emission reductions. We further test the robustness of these results by evaluating the model across a variety of scenarios that could influence the impact of temporal flexibility.

**Figure 9:**
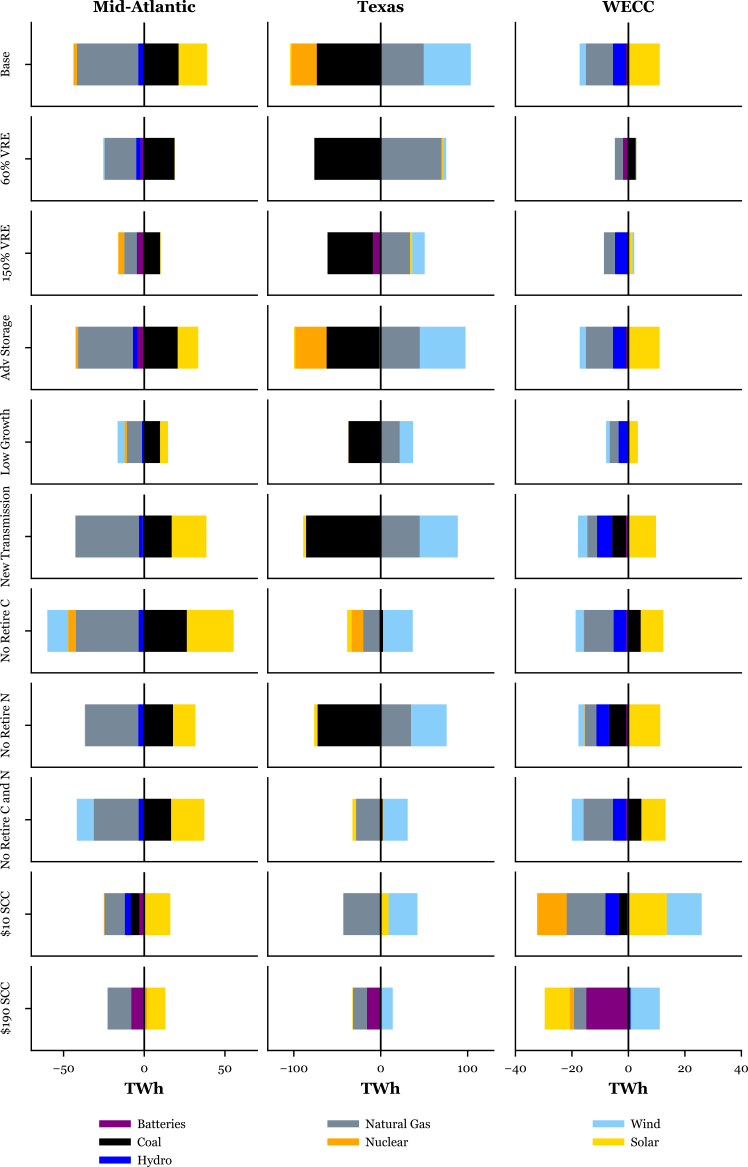
Difference in generation per technology type between the “flexibility” and ‘no flexibility” scenarios Stacked bar charts represent the difference in total generation per technology type. A positive (negative) value indicates larger generation in the “flexibility” (“no flexibility”) scenario. Each row is a sensitivity analysis scenario, and each column is a region. Note the different *x* axis scales per column.

These sensitivity scenarios focus on (1) assessing alternative technologies that could also provide similar flexibility, (2) load forecasts for data centers, (3) technology-related policies that change the generation portfolio of a region, and (4) carbon policies in the form of a social cost of carbon. These are described in Table 1. We evaluate each sensitivity scenario for each region under two cases: “no flexibility” and “flexibility” with a 24-h shifting horizon and 100% share of flexible workload. These represent the two extreme cases of systems with and without flexibility. Tables 2 and 3 show the percentage reduction in cost and emissions, respectively, between these two extremes. For ease of reference, we also show the change in the generation mix in Figure 9 (data on the change in capacity investments and retirements can be found in Note S10 and Note S11, respectively).

**Table 1:** Sensitivity analysis scenarios

Category	Sub-category	Description	Base value	Sensitivity scenarios
Alternative technologies	transmission builds	constraint on new intraregional lines	no new builds	×2 of existing capacity
–	storage costs	battery cost projection based on NREL ATB (Annual Technology Baseline)	moderate ATB	advanced ATB
Data center forecast	data center forecast	forecasted data center load per region	high load forecast	low load forecast
Generation tech policies	VRE (Variable Renewable Energy) penetration	fraction of optimal VRE build-outs realized	no limit	60%, 150% of optimal VRE realized
–	non-retirement of baseload	baseload plants are forced not to retire	no constraints	coal, nuclear, both don’t retire
Carbon policies	social cost of carbon (SCC)	implementation of an SCC per MT of CO_2_	$0 SCC	$10, $190 SCC

**Table 2:** System cost of a “no flexibility” (No Flex) and a 24-h shifting horizon, 100% flexible workload system (Flex) under each sensitivity scenario

	Texas			Mid-Atlantic			WECC		
–	No Flex	Flex	% Change	No Flex	Flex	% Change	No Flex	Flex	% Change
Base model	16.13	15.28	5.29	30.97	29.56	4.55	24.48	23.80	2.76
60% VRE penetration	16.21	15.49	4.45	31.11	29.72	4.45	24.59	24.02	2.29
150% VRE penetration	18.14	16.58	8.55	31.59	29.97	5.14	24.93	24.12	3.24
Advanced storage	16.14	15.28	5.29	30.88	29.52	4.40	24.48	23.80	2.76
Low data center growth	13.17	12.82	2.66	26.88	26.27	2.28	20.87	20.57	1.41
New intraregional transmission allowed	15.76	14.87	5.63	30.91	29.44	4.77	24.39	23.72	2.72
Non-retirement of coal	16.14	15.46	4.18	31.24	30.21	3.32	24.59	23.96	2.58
Non-retirement of nuclear	16.13	15.31	5.12	31.08	29.68	4.50	24.51	23.84	2.72
Non-retirement of coal and nuclear	16.18	15.59	3.63	31.41	30.46	3.03	24.62	24.01	2.48
$10 social cost of carbon	17.07	16.01	6.21	33.19	31.63	4.71	26.49	25.73	2.88
$190 social cost of carbon	24.96	22.37	10.35	53.86	52.00	3.45	36.62	34.84	4.84

**Table 3:** System emissions of a “no flexibility” (No Flex) and a 24-h shifting horizon, 100% flexible workload system (Flex) under each sensitivity scenario

	Texas			Mid-Atlantic			WECC		
–	No Flex	Flex	% Change	No Flex	Flex	% Change	No Flex	Flex	% Change
Base model	149.67	92.01	38.52	280.58	292.36	−4.20	237.03	232.82	1.78
60% VRE penetration	163.58	111.86	31.62	299.63	315.16	−5.18	269.04	270.95	−0.71
150% VRE penetration	93.94	50.88	45.83	235.31	245.13	−4.17	192.82	190.24	1.34
Advanced storage	138.10	92.00	33.38	280.30	289.97	−3.45	237.03	232.82	1.78
Low data center growth	106.97	76.16	28.80	238.79	246.48	−3.22	200.40	198.98	0.71
New intraregional transmission allowed	152.27	78.97	48.14	281.41	287.98	−2.33	234.22	226.77	3.18
Non-retirement of coal	163.77	158.30	3.33	317.93	332.20	−4.49	262.35	262.84	−0.19
Non-retirement of nuclear	139.58	76.18	45.42	273.84	283.66	−3.59	233.87	224.96	3.81
Non-Retirement of coal and nuclear	158.02	148.75	5.87	305.44	312.97	−2.46	259.04	259.09	−0.02
$10 social cost of carbon	77.90	65.22	16.27	185.65	178.12	4.06	144.99	137.67	5.05
$190 social cost of carbon	28.55	21.61	24.32	62.12	56.42	9.18	30.75	29.02	5.63

Overall, we find that the main results still apply, albeit at varying levels. The change in the % difference in the cost and emissions between the “no flex” and “flex” systems relative to the baseline depends on whether the sensitivity scenario favors renewables or baseload plants. For example, a baseload-favoring scenario of only 60% VRE penetration results in lower cost (4.45% in Texas vs. 5.29% baseline) and emission reductions (31.62% in Texas vs. 38.52% baseline) when a system has data center flexibility. Compare this to the same case with 150% VRE penetration where the cost and emission reductions for Texas increase to 8.55% and 45.83%, respectively. Similarly, if coal is forced to stay in the system and is not retired, then the emission impact worsens (3.33% in Texas vs. 38.52% baseline).

In the case where nuclear plants are forced to stay in the system while coal plants can be retired (i.e., “no retire” N scenario), temporal flexibility also moves toward favoring baseload generation. However, the emission impact improves as nuclear generation replaces coal when there is flexibility in both Texas and WECC and replaces natural gas in the Mid-Atlantic (Figure 9). This emphasizes an important point: data center temporal flexibility’s tendency to favor baseload generation does not always lead to an increase in emissions. If the most cost-efficient baseload technology is clean (e.g., nuclear), then temporal flexibility can still improve emission outcomes in these cases. Note, however, that flexibility makes it cost efficient to retire nuclear plants, and, so, nuclear-favoring data flexibility has to be accompanied with policies that keep nuclear plants in the system to realize these results.

The economic outlook of grid-scale battery storage has a limited effect on the role of data center flexibility. Although both serve to shift energy temporally and provide similar system benefits, data center flexibility in our model acts as a cost-free resource, whereas battery storage requires investment, even in the optimistic (i.e., advanced ATB) case where costs decline much faster. While the impact of temporal flexibility on system cost is minimally affected by additional intraregional transmission, greater transmission expansion leads to larger emission reductions. Enhanced transmission capacity enables more cost-effective integration of renewables, which, in turn, increases the value of temporal flexibility. This reflects findings of Senga et al.^25^^,^^30^ Finally, assigning a cost to carbon emissions through a social cost of carbon results in a reduction in emissions in all regions, including the Mid-Atlantic.

## Discussion

Data centers are projected to account for a substantial share of US electricity demand in the coming years. This study examines how the temporal flexibility of data center load can alter power systems in response to this rapid demand growth. Specifically, we analyzed how flexibility affects grid operations, investment and retirement decisions, system costs, and emission outcomes.

Recent developments in both regulatory approaches and industry practices show that there is growing attention to the challenges of accommodating large loads within existing grid and market frameworks. In particular, the growth in data center demand has been accompanied by increasingly long interconnection queues, which create significant barriers to timely grid connection. While this process represents a major bottleneck for data center owners, the interconnection study process remains essential to ensure that the addition of large, concentrated loads does not compromise grid stability. Recently, the US Department of Energy (DOE) issued a proposal to expedite the interconnection process, emphasizing that “curtailability” and “dispatchability” could enable system operators to integrate large loads, such as data centers, more efficiently.^31^

This initiative builds upon precedents from utilities, state regulators, and system operators that have introduced curtailment requirements for qualifying large loads. For instance, Texas Senate Bill 6 was recently enacted, granting the system operator authority to curtail loads of 75 MW or greater during peak load-shed events.^32^ Similarly, PJM’s proposed non-capacity backed load (NCBL) initially mandated curtailment for loads above 50 MW, though subsequent opposition from data center operators resulted in a voluntary framework instead.^33^^,^^34^ Southwest Power Pool (SPP) has adopted a comparable approach, proposing to fast-track interconnection studies for large loads that can demonstrate curtailability under its Conditional High Impact Large Load (CHILL) program.^35^

While our study does not directly model grid strain or reliability impacts, our results demonstrate that data center flexibility consistently leads to cost benefits. Current debates around data center interconnection, such as opposition to PJM’s NCBL proposal, often center on concerns about potential revenue losses from curtailment. Our analysis suggests that incorporating controlled flexibility into data center operations can offset substantial system costs. Flexible data centers can adjust their load profiles in response to system conditions, enabling more efficient power system operations. We find that the cost-optimal load-shifting strategy tends to flatten the net load curve by reducing demand during peak hours and shifting it toward periods of low net load. This operational adjustment reduces reliance on peaker plants and enables better utilization of low-cost renewable energy, resulting in lower system costs compared to inflexible demand. The magnitude of these savings depends on both the share of flexible workload and the shifting horizon, with more flexibility leading to lower cost. By reframing flexibility not as a constraint but as an asset, system operators and policymakers can design market or incentive mechanisms that align private incentives with broader system efficiency while ensuring grid stability.

A parallel question concerns how market-based mechanisms can complement flexibility by internalizing the environmental costs of data center operations. Carbon commitments from large technology companies like Google, Meta, and NVIDIA see temporal flexibility as an important tool to meet emission goals.^22^^,^^36^ However, our results show that the magnitude and direction of emission impacts are context dependent and vary with the regional generation mix, renewable penetration, transmission availability, and policies that keep certain technologies like coal and nuclear online (see Table 3). In systems with abundant and cost-competitive renewables, flexibility supports decarbonization by aligning demand with clean energy availability. However, in fossil-heavy grids, flexibility can increase emissions by increasing coal generation. Complementary policies that accelerate renewable deployment or lower clean energy costs are needed alongside flexibility incentives to ensure that the emission-reduction potential of data center temporal flexibility is fully realized. A way to do this is through the implementation renewable mandates. We have shown through the sensitivity analysis that renewable mandates that increase the share of VRE resources are valuable, but the impact on emissions may still not be enough given the relative cost efficiency of coal (see Table 3). Alternatively, implementing a carbon price is more efficient, which we similarly show to have a positive impact when coupled with data center flexibility. Even modest carbon prices can lead to a reversal of the base emission results for the Mid-Atlantic, with an emission reduction of 9.18% with temporal flexibility relative to having no flexibility.

Data center flexibility also has important implications for long-term capacity investment and retirement decisions. These impacts depend on the existing generation mix, renewable resource availability, and the evolving costs of clean energy technologies. In general, temporal flexibility encourages investment in wind and solar by shifting demand to hours with low marginal costs, which often align with high renewable production. In high renewable systems like Texas, where renewables comprise 50% of generation, flexibility leads to increased investment in VRE capacity, accelerated retirement of baseload capacity, and increased reliance on flexible thermal generation to manage intermittency. In contrast, in regions with lower renewable shares, such as the Mid-Atlantic, flexibility can increase the utilization of baseload plants like coal, even if some retirements still occur.

Overall, we find that the rapid growth in data center load has significant implications for power system planning and operations. Its inherent capability to temporally shift load can be used to mitigate the costs and infrastructure needs associated with this increased demand. To ensure that this flexibility supports decarbonization, it must be deployed alongside strong clean energy policies that accelerate renewable deployment. When aligned with such policies, data center flexibility can act as a valuable grid asset, lowering peak capacity, reducing cost, and facilitating the integration of variable renewable resources.

### Limitations of the study

Our model optimizes a representative year of operation where the investment, dispatch, and data shifting decisions are made by a centralized entity. Decentralized decision-making of carbon-aware loads is not accounted for but was studied by Jiang et al.^19^ We assume that data center load is constant and that it can freely shift its load within the hourly horizon without the need for ramping and without disruptions to the operations. The costs associated with data center load shifting are not modeled, as we focus on AI training workloads whose advancement or delay can be rescheduled, with essentially no impact on user experience. The direct costs of shifting such training tasks are typically negligible or highly application-specific, which makes them difficult to quantify. The stochastic variability of VRE and load is not considered by assuming exogenous, deterministic time-series inputs. We also assume that there are no restrictions on the amount of new capacity that can be built and that this capacity can be built by the model year. Finally, our findings are based on three US regional systems: Texas, the Mid-Atlantic, and WECC. The direction and magnitude of flexibility impacts tend to vary across regions depending on renewable availability, fuel prices, and institutional design. Extrapolating these results to other regions or countries should, therefore, be done with caution and supported by additional modeling that reflects local resource endowments, policy environments, and system characteristics. The model can then be thought of as a stylized and idealized US power system with data centers.

## Resource availability

### Lead contact

Requests for further information and resources should be directed to and will be fulfilled by the lead contact, Juan Ramon L. Senga (jsenga@mit.edu).

### Materials availability

No reagents were generated in this study.

### Data and code availability

• Data necessary to replicate the results in the paper are provided in: https://github.com/JRLSenga/DataCenter_TemporalFlex.
• Code necessary to replicate the results in the paper is provided in: https://github.com/JRLSenga/DataCenter_TemporalFlex.
• Any additional information required to analyze the data reported in this paper is available from the lead contact upon request. On request, the authors can provide the files to reproduce all numbers and figures.

## Acknowledgments

The authors would like to acknowledge J.E. Parsons, J. Hodge, G. Metcalf, D. Deka, R. Field, and M. Andreae for their insights and discussion on models, assumptions, and policy implications. All views expressed in this paper are those of the authors and do not necessarily reflect the views of acknowledged individuals or affiliated institutions. This research was supported by funding from the Future Energy Systems Center of the MIT Energy Initiative (MITEI).

## Author contributions

Conceptualization, J.R.L.S., S.W., and C.R.K; data curation, J.R.L.S. and S.W.; software and creation of GenX model version, J.R.L.S. and S.W.; methodology, J.R.L.S. and S.W.; investigation, J.R.L.S., S.W., and C.R.K.; visualization, J.R.L.S. and S.W.; writing – original draft and writing – review & editing, J.R.L.S., S.W., and C.R.K.

## Declaration of interests

The authors declare no competing interests.

## STAR★Methods

### Key resources table

**Table:** 

REAGENT or RESOURCE	SOURCE	IDENTIFIER
**Software and algorithms**		

GenX Software	Authors	https://github.com/JRLSenga/DataCenter_TemporalFlex

**Deposited Data**		

Input and Output Data	Authors	https://github.com/JRLSenga/DataCenter_TemporalFlex

### Method details

#### GenX capacity expansion model

The analysis uses the Capacity Expansion model, GenX. Details and documentation on the GenX model can be found in https://genxproject.github.io/GenX.jl/dev/. The specific implementation used for this work can be found in the Supplementary Code: https://github.com/JRLSenga/DataCenter_TemporalFlex. GenX is a least-cost mixed integer linear programming (MILP) model that co-optimizes generation and transmission investments and dispatch decisions among pre-defined zones within the power system. The optimization accounts for capital, operational, and fuel costs, generator technical operating characteristics, capacity factors for renewables, and demand information. The objective is to minimize annual system cost, which is the sum of investment in generation and storage, fixed and variable operating and maintenance costs, new transmission investment costs, fuel and startup costs, accounting for tax credits and other incentives, if any. GenX assumes a representative year of operation and perfect foresight of hourly demand and capacity factor data for renewables supply in its dispatch decisions. We source input data from PowerGenome^37^ which is a data processing software that aggregates data from publicly available sources such as NREL’s ATB for cost data,^38^ NREL’s EFS for demand data^39^^,^^40^ and EIA’s Form-860^41^ for existing generator data. Details on all inputs can be found in the Supplementary Text.

#### Modeling data center temporal flexibility

We modified the GenX code-base to include temporal flexibility of data centers. GenX already has the capability to represent flexible demand resources built into the base model, but we included constraints to constrain the amount that can be shifted to and from an hour, given the capacity of a data center. For our model, data centers that can shift load temporally are modeled similarly to storage assets that can “store” demand from each hour and must be deployed elsewhere within the shifting horizon.unknown.

Consider a representative data center with capacity C and data center load Lt at hour t. Temporal flexibility is defined by two parameters: First is the shifting horizon h, which is the number of hours before or after the originally scheduled hour in which data center load can be deferred and advanced. Second, the share of flexible workload s, (0 ≤ s ≤ 1), which is the fraction of total data center demand that can be shifted to other hours.

Let $Y_{t}\in R$ be the amount of data center demand that is yet to be satisfied at hour t. A positive value means that there exists demand that is delayed, and a negative value means that demand has been advanced. Let St ≥ 0 be the amount of shifted data center load that is satisfied at hour t. Finally, let Dt ≥ 0 be the amount of data center load that was originally allocated to hour t but is deferred and will be satisfied in a future hour t′, where t < t′ ≤ t + h. We include the following constraint to model data center flexibility.

Data Center Load Balancing Constraint: the amount of data center demand that is yet to be satisfied is equal to the amount from the previous hour, less what is satisfied in the current hour, plus any deferrals.

(Equation 1) $$Y_{t}=Y_{t-1}-S_{t}+D_{t}$$

*Maximum Time to Delay Demand Constraint*: The amount of data center load that is satisfied in the next *h* hours from time *t* must be greater than or equal to the amount that is yet to be satisfied by time *t*.

(Equation 2) $$\overset{t+h}{\underset{i=t+1}{\sum }}S_{i}\geq Y_{t},\forall t$$

*Maximum Time to Advance Demand Constraint*: The amount of data center load that is deferred in the next *h* hours from time *t* must be greater than the advanced demand (negative of *Y*_*t*_) in hour *t*.

(Equation 3) $$\overset{t+h}{\underset{i=t+1}{\sum }}D_{i}\geq -Y_{t},\forall t$$

*Maximum amount of demand Deferred Constraint*: The amount of demand that can be deferred in each hour must be less than the share of flexible workload.

(Equation 4) $$D_{t}\leq sL_{t},\forall t$$

*Maximum Data Center Load Satisfied*: Shifted data center load that is satisfied during an hour must be less than or equal to the capacity of the data center net of the deferred demand.

(Equation 5) $$S_{t}\leq C-L_{t}+D_{t}$$
