# Supplementary material for: Flexible data centers reduce power system costs but can increase emissions
Source: iScience. 2026 Jun 26;29(7):116497. doi: 10.1016/j.isci.2026.116497 (PMC13377865; doi:10.1016/j.isci.2026.116497)
Supplement: Document S1. Figures S1–S11 and Tables S1–S6 [file mmc1.pdf]

iScience, Volume *29*

Supplemental information

Flexible data centers reduce power system costs but can increase emissions

Juan Ramon L. Senga, Shen Wang, and Christopher R. Knittel

S1 Input Assumptions

S1.1 Regions and Load

Table S1 shows the number of zones per region along with the total and hourly data center load, and the total non-data center load per zone. Zones are IPM regions from the EPA. Data center load is sourced from [1] while non-data center load is sourced from [2] who source the base hourly demand from NREL's EFS [3]. We use the "High Growth" scenario as our base case.

Table S1: Base Data Center and Non-Data Center Load per Model Zone (in MWh)

Region	Zone	Zone Number	Data Center Load (No Growth)	MWh per Hour (Base)	Data Center Load (Base)	Non-Data Center Load	Total Load (Base)	Share of Data Center Load
Texas	ERC_REST	1	19,196,070	15,415	135,033,849	399,018,968	534,052,817	25.3%
Texas	ERC_WEST	2	852,970	685	6,000,174	17,730,244	23,730,418	25.3%
Texas	ERC_PHDL	3	268,607	216	1,889,506	5,583,406	7,472,912	25.3%
Mid-Atlantic	PJM_AP	1	1,838,296	600	5,253,312	56,335,623	61,588,935	8.5%
Mid-Atlantic	PJM_ATSI	2	1,415,551	2,634	23,076,374	88,176,254	111,252,627	20.7%
Mid-Atlantic	PJM_Dom	3	30,409,937	9,605	84,137,386	88,425,299	172,562,685	48.8%
Mid-Atlantic	PJM_EMAC	4	5,470,400	1,280	11,212,385	170,081,177	181,293,562	6.2%
Mid-Atlantic	PJM_PENE	5	678,669	221	1,939,439	20,798,210	22,737,648	8.5%
Mid-Atlantic	PJM_SMAC	6	107,757	10	90,179	67,240,255	67,330,434	0.1%
Mid-Atlantic	PJM_WMAC	7	1,394,599	455	3,985,355	42,738,269	46,723,625	8.5%
Mid-Atlantic	PJM_West	8	15,408,103	6,131	53,703,310	170,446,981	224,150,291	24.0%
WECC	WEC_CALN	1	3,618,438	568	4,977,247	94,177,173	99,154,420	5.0%
WECC	WEC_LADW	2	2,710,231	426	3,727,987	70,539,254	74,267,241	5.0%
WECC	WEC_SDGE	3	899,730	141	1,237,600	23,417,298	24,654,898	5.0%
WECC	WECC_SCE	4	2,838,787	446	3,904,820	73,885,197	77,790,016	5.0%
WECC	WECC_MT	5	641,553	59	518,599	16,650,987	17,169,585	3.0%
WECC	WEC_BANC	6	510,699	80	702,479	13,291,990	13,994,469	5.0%
WECC	WECC_ID	7	141,588	13	118,052	24,698,492	24,816,543	0.5%
WECC	WECC_NNV	8	1,038,416	149	1,303,616	10,911,131	12,214,747	10.7%
WECC	WECC_SNV	9	2,827,400	405	3,549,487	29,708,849	33,258,336	10.7%
WECC	WECC_UT	10	3,030,388	358	3,133,353	36,427,790	39,561,143	7.9%
WECC	WECC_PNW	11	11,987,277	8,449	74,011,855	165,076,398	239,088,252	31.0%
WECC	WECC_CO	12	1,886,168	310	2,715,211	69,022,399	71,737,610	3.8%
WECC	WECC_WY	13	1,626,323	146	1,278,063	23,664,710	24,942,773	5.1%
WECC	WECC_AZ	14	6,871,657	7,242	63,441,221	85,613,629	149,054,850	42.6%
WECC	WECC_NM	15	612,040	58	506,741	40,742,002	41,248,743	1.2%
WECC	WECC_IID	16	53,403	8	73,457	1,389,919	1,463,376	5.0%

The EPRI report [4] indicates what % of each state's 2023 electricity demand was for Data Centers. We assume that without data center growth, load will have the same % share of data center load in 2030. We then calculate the additional data center load on top of the % share in the base case. We provide an illustrative example for Texas Zone 1:

1. 4.59% of Texas' load in 2023 is for Data Centers.
2. 2030 NREL EFS Demand for Texas Zone 1 is 418.2 TWh. 418 TWh $\times$ 4.59% = 19.2 TWh of Base Data Center Load in 2030
3. 418.2 TWh $-$ 19.2 TWh = 399 TWh of non-Data Center Load in 2030
4. There is a high growth forecast of 25.28% of Texas 2030 load will come from Data Centers
5. 399 TWh / (100% $-$ 25.28%) = 534 TWh total Texas Zone 1 Load.
6. 534 TWh $\times$ 25.28% = 135 TWh of Data Center load in 2030.
7. Since we assume that data center load is constant per hour, we divide the 135 TWh by 8760 hours.

S1.2 Generators

Our model includes existing capacity generators as well as a set of new technologies that can be deployed. Existing generation capacity is sourced from EIA Form-860 and aggregated through PowerGenome [2]. Details can be found in Table S2. Investment, operating, and maintenance costs for new generators can be found in Table S3. Fixed O&M costs, CAPEX, and WACC for new capacity are taken as average values from NREL ATB 2022 from the years 2023 to 2030 [5]. The investment costs vary based on regional multipliers. Meanwhile, cost assumptions for existing plants use the basis year 2020, with variation assumptions from PowerGenome depending on the start year of operation. Production and tax credits associated with the Inflation Reduction Act are also implemented in the model.

Table S2: Capacity of Existing Generators per Technology in each Region (in GW)

Technology	Mid-Atlantic	Texas	WECC
Batteries	0.25	4.40	13.69
Conventional Hydroelectric	3.35	0.54	50.25
Conventional Steam Coal	39.24	13.63	22.08
Hydroelectric Pumped Storage	5.21	0.00	5.05
Natural Gas Fired Combined Cycle	55.90	41.75	52.75
Natural Gas Fired Combustion Turbine	22.26	11.16	23.57
Nuclear	22.80	5.12	7.42
Onshore Wind Turbine	5.97	34.05	32.22
Solar Photovoltaic	10.79	20.83	38.27

Table S3: New Technology Investment and Operation Cost Assumptions in 2030

	Capex	Capital Recovery Period	WACC	Investment Cost	Fixed O&M	Variable O&M
	($/MW)	(years)		($/MW-yr)	($/MW-yr)	($/MWh)
Natural Gas Combined Cycle	932,813	15	3.56%	81,708	28,000	2
Solar Photovoltaic	913,819	20	2.50%	58,794	22,623	-
Onshore Wind Turbine	1,131,578	20	3.06%	76,816	40,367	-
Battery	250,489	20	2.50%	16,116	6,262	-

S1.3 Transmission

We source current transfer capabilities per line between each IPM zone from the EPA's Power Sector Modeling Platform v6—2021 Summer Reference Case [6]. We assume a pipeline flow model such that the amount of transmission that can flow between two zones is only restricted by the capacity of the line.

S1.4 Net Imports

Texas and WECC are fairly isolated as model regions within the continental U.S.. The impacts of electricity exchange with neighboring regions on these two regions via transmission lines is therefore minimal. However, the Mid-Atlantic is extensively connected to other neighboring regions such as the Midwest, Southeast, and New York. To account for this in the model, we sourced hourly net import data for the Mid-Atlantic from EIA's Grid Monitor Dashboard for 2022 (https://www.eia.gov/electricity/gridmonitor/dashboard/electric_overview/US48/

US48). Within the dataset, Mid-Atlantic's (MIDA) net imports are aggregated to hourly exchange with CAR (Carolinas), MIDW (Midwest), NY (New York), and TEN (Tennessee). To allocate the net import to model zones, we first determine whether the model zone has existing transmission capacity with the EIA regions. If there is, we calculate the percentage allocation as the total load of the model zone divided by the total load of all model zones connected to the region (see Table S4). Each model zone's net import is thus the hourly net import from the EIA data set multiplied by this allocation percentage.

Table S4: Net Import Allocation Percentage

	Zone Number	CAR	MIDW	NY	TEN
PJM_AP	1	0%	0%	0%	0%
PJM_ATSI	2	0%	0%	0%	0%
PJM Dom	3	43%	0%	0%	0%
PJM_EMAC	4	0%	0%	89%	0%
PJM_PENE	5	0%	0%	11%	0%
PJM_SMAC	6	0%	0%	0%	0%
PJM_WMAC	7	0%	0%	0%	0%
PJM_West	8	57%	100%	0%	100%

S1.5 CO_2 Emissions Factors

Emission factors are available for Natural Gas and Coal. CO_2 is generated per MMBtu of fuel consumed. We assume 0.09552 mtCO_2/MMBtu and 0.05306 mtCO_2/MMBtu for coal and natural gas, respectively. Table S5 shows the average heat rates for existing generators.

Table S5: Average Heat Rates of Existing Generators (in MMbtu/MWh)

	Mid-Atlantic	Texas	WECC
Conventional Steam Coal	12.27	11.28	10.97
Natural Gas Fired Combined Cycle	8.19	8.69	8.07
Natural Gas Fired Combustion Turbine	13.12	12.10	12.08
Nuclear	10.45	10.45	10.45

S1.6 Supply Curves

Supply curves for renewables are sourced from PowerGenome [2], who source the data from Vibrant Clean Energy's data sets [7].

S1.7 Fuel Costs

Fuel costs are sourced from EIA's Annual Energy Outlook (AEO) 2022 for the year 2030. The individual zones are matched to the AEO regions through PowerGenome. Fuel cost information can be found below in Table S6.

S2 Capacity Retirements

Fig. S1 shows the impact of flexible data centers on retirement decisions for nuclear, coal, and natural gas generators across the three regions. In the Mid-Atlantic and WECC, retirement

Table S6: Fuel Cost (in $/MMbtu)

Model Region	Fuel	AEO Region	Fuel Name	Model Zone	$/MMBtu
Mid-Atlantic	Coal	South Atlantic	south_atlantic_coal	PJM_AP, PJM_Dom	2.40
		East North Central	east_north_central_coal	PJM_ATSI, PJM_West	1.84
		Middle Atlantic	middle_atlantic_coal	PJM_EMAC, PJM_PENE, PJM_SMAC, PJM_WMAC	2.25
	Natural Gas	South Atlantic	south_atlantic_naturalgas	PJM_AP, PJM_Dom	4.10
		East North Central	east_north_central_naturalgas	PJM_ATSI, PJM_West	3.41
		Middle Atlantic	middle_atlantic_naturalgas	PJM_EMAC, PJM_PENE, PJM_SMAC, PJM_WMAC	3.19
	Uranium	South Atlantic	south_atlantic_uranium	PJM_Dom	0.71
		East North Central	east_north_central_uranium	PJM_ATSI, PJM_West	0.71
		Middle Atlantic	middle_atlantic_uranium	PJM_EMAC, PJM_SMAC, PJM_WMAC	0.71
Texas	Coal	West South Central	west_south_central_coal	ERC_REST	1.73
	Natural Gas	West South Central	west_south_central_naturalgas	ERC_REST	3.49
	Uranium	West South Central	west_south_central_uranium	ERC_REST, ERC_PHDL, ERC_WEST	0.71
WECC	Coal	Mountain	mountain_coal	WECC_AZ, WECC_CO, WECC_MT, WECC_NM, WECC_NNV, WECC_UT, WECC_WY	1.55
		Pacific	pacific_coal	WECC_PNW, WECC_SCE	2.02
	Natural Gas	Mountain	mountain_naturalgas	WECC_AZ, WECC_CO, WECC_ID, WECC_MT, WECC_NM, WECC_NNV, WECC_SNV, WECC_UT, WECC_WY	4.00
		Pacific	pacific_naturalgas	WECC_IID, WECC_PNW, WECC_SCE, WEC_BANC, WEC_CALN, WEC_LADW, WEC_SDGE	3.88
	Uranium	Mountain	mountain_uranium	WECC_AZ	0.71
		Pacific	pacific_uranium	WECC_PNW, WEC_CALN	0.71

decisions appear largely insensitive to data center flexibility across all three fuel types, although coal and natural gas show an increase in retirements for the Mid-Atlantic (Fig. S1B) and WECC (Fig. S1I, respectively. Nuclear retirements for both regions (Fig. S1A, Fig. S1G) and natural gas retirements for the Mid-Atlantic (Fig. S1C) remain similar regardless of flexibility levels. This suggests that the generation mix and system constraints in these regions limit the ability of flexible demand to displace firm capacity.

In contrast, Texas sees different retirement patterns. As both the share of flexible workload and the shifting horizon increase, significant generator retirements are observed, particularly for nuclear (Fig. S1D) and coal (Fig. S1E) resources. At high flexibility levels and long shifting horizons (e.g., $\geq$ 80% flexible workload and 24-hour shifting horizon), nuclear and coal retirements approach or exceed 80% and 90%, respectively. This indicates that flexible data center demand in Texas has a capacity substitution effect, particularly for baseload resources. This is due to the region's high penetration of high-quality renewables. Natural gas retirements in Texas (Fig. S1F) remain low overall, with only marginal increases under the highest flexibility levels.

S3 Capacity Investments

Fig. S2, S3, and S4 show the effect of flexible data center operations on new capacity investments in the Mid-Atlantic, Texas, and WECC, respectively.

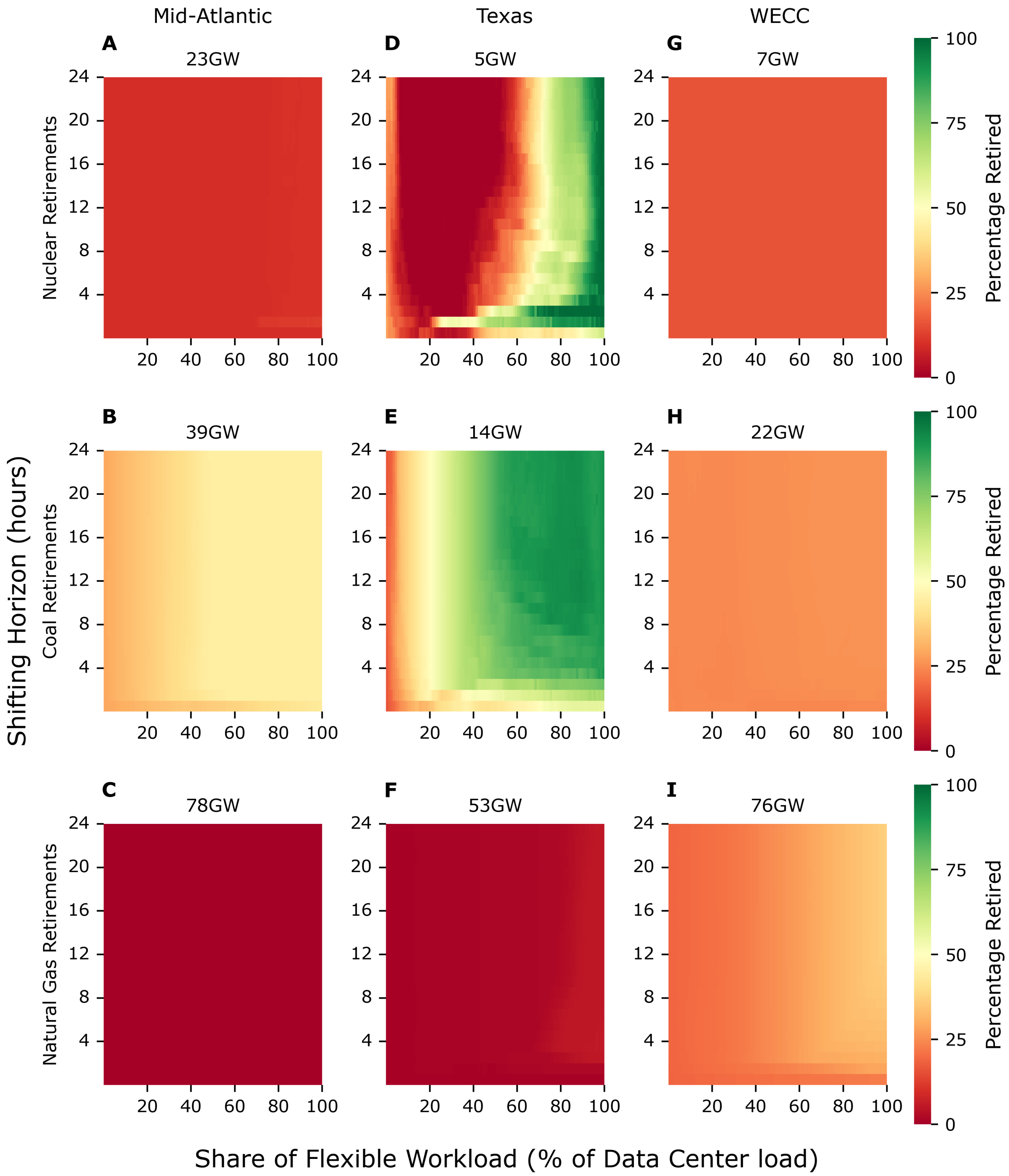

Figure S1: Percentage Retirement per Technology for each Region. Panels show the percentage of existing capacity retired for nuclear **(A, D, G)**, coal **(B, E, H)**, and natural gas **(C, F, I)** across combinations of data center shifting horizon (1 to 24 hours) and flexible workload share (1% to 100%). Values displayed at the top of each heatmap indicate the initial installed capacity for the corresponding technology in each region.

In the Mid-Atlantic, solar (Fig. S2A) investments increase significantly with higher levels of data center flexibility, particularly when both the share of flexible workload exceeds 60% and the shifting horizon extends beyond 2 hours. Under these conditions, solar capacity reaches over 52 GW. This reflects the ability of the system to align flexible data center demand with solar output. This increases the value of solar in balancing load within a day, which leads to

more investments. Investments in wind capacity (Fig. S2B), in contrast, remain unchanged across the different combinations of flexibility. This suggests that the temporal characteristics of flexible demand do not substantially affect wind investment decisions in the Mid-Atlantic. Battery investments (Fig. S2C) remain relatively limited, with total new capacity not exceeding 2 GW. Notably, battery deployment decreases once the flexible workload share exceeds 60%. This decline can be attributed to functional competition between batteries and flexible data center loads, as both serve similar roles in providing temporal flexibility to the power system. As flexible data center operations become more prominent, they can displace the need for additional storage by shifting load in response to system conditions. Investments in new natural gas capacity (Fig. S2D) decrease as data center flexibility increases. With high levels of both the share of flexible workload and long shifting horizons, natural gas investment drops from over 14 GW to below 6 GW, indicating that flexible demand can substitute for peaking gas capacity by reducing peak load and system ramping needs.

In Texas, a higher level of data center flexibility leads to an increase in wind investments from approximately 46 GW to over 58 GW (Fig. S3B). Solar capacity (Fig. S3A) shows a more modest and stable pattern with only a slight increase from 19 GW to 22.5 GW. The flatter gradient suggests that while solar remains valuable, its incremental benefit diminishes in the presence of high data center flexibility. This is due to the temporal mismatch between peak solar output and peak system stress in Texas. Battery and natural gas investments (Fig. S3C, S3D) remain negligible across the entire flexibility space, with capacities barely exceeding 0.05 GW. Thus, in Texas, data center temporal flexibility can strongly incentivize wind deployment, supporting a more renewable-heavy system configuration. Similar to PJM, this indicates that flexible data center operations are effectively substituting for both short-duration storage and fast-ramping thermal resources.

In WECC, both solar and wind capacity exhibit noticeable increases as data center flexibility increases. Solar investments (Fig. S4A) increase steadily from 38 GW to over 43 GW, particularly when the share of flexible workload exceeds 40% and the shifting horizon is greater than 8 hours. Similarly, wind capacity (Fig. S4B) shows an upward trend, growing from 13 GW to 14 GW under higher flexibility. Just like in the Mid-Atlantic and Texas, these patterns also suggest that flexible data center demand in WECC increases the economic viability of variable renewables. In contrast, there are no new battery investments (Fig. S4C) across all flexibility scenarios. Natural gas (Fig. S4D) investments are small even without flexibility at around 1.75 GW, and decline to almost no investments as flexibility increases. This indicates that the load flexibility is sufficient to meet system balancing needs, diminishing the marginal value of new natural gas plants.

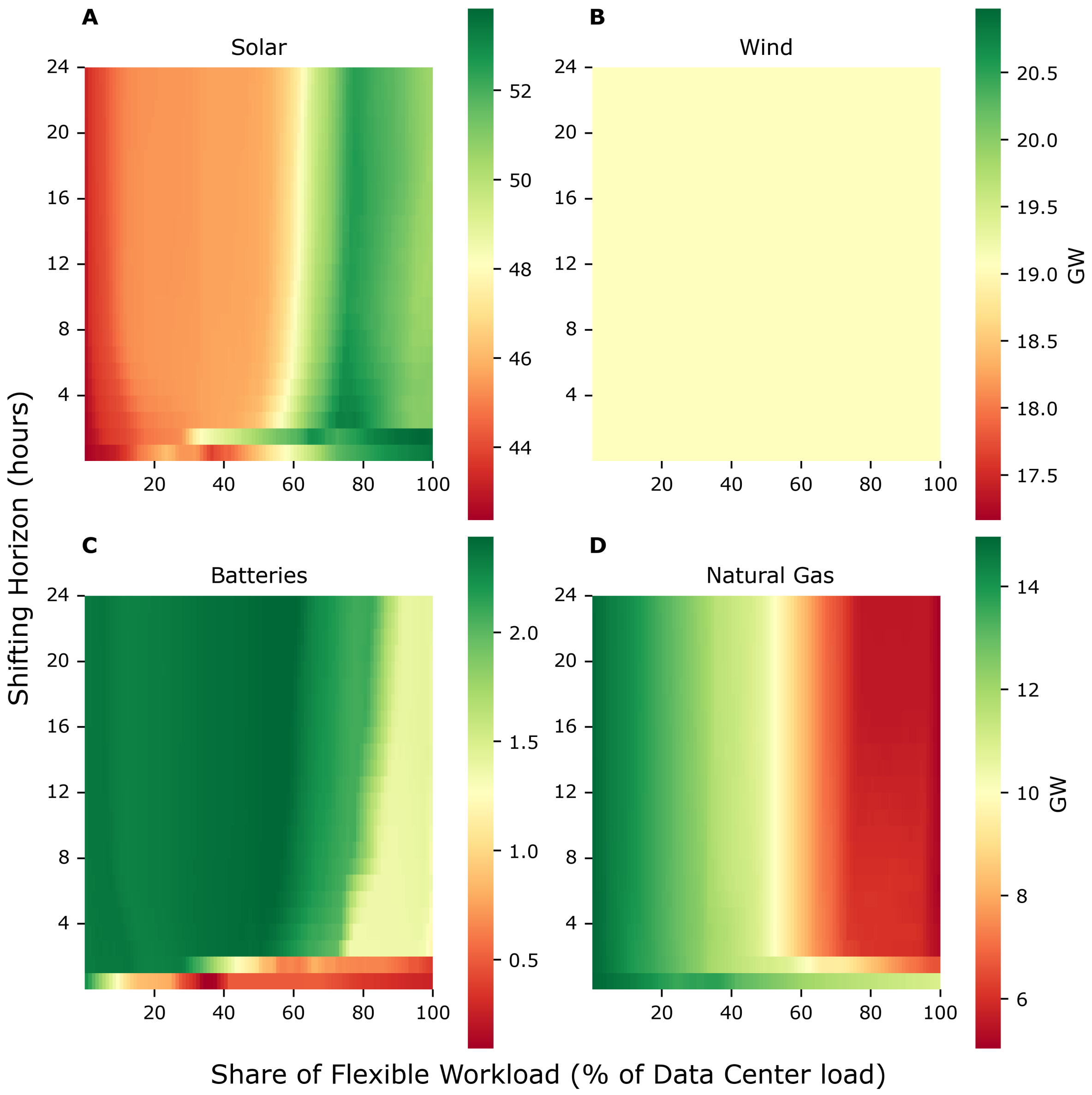

Figure S2: Capacity Investments per Technology in the Mid-Atlantic. Panels show new capacity additions for solar **(A)**, wind **(B)**, batteries **(C)**), and natural gas **(D)** across combinations of data center shifting horizon (1 to 24 hours) and flexible workload share (1% to 100%). Note that color scales vary across subplots.

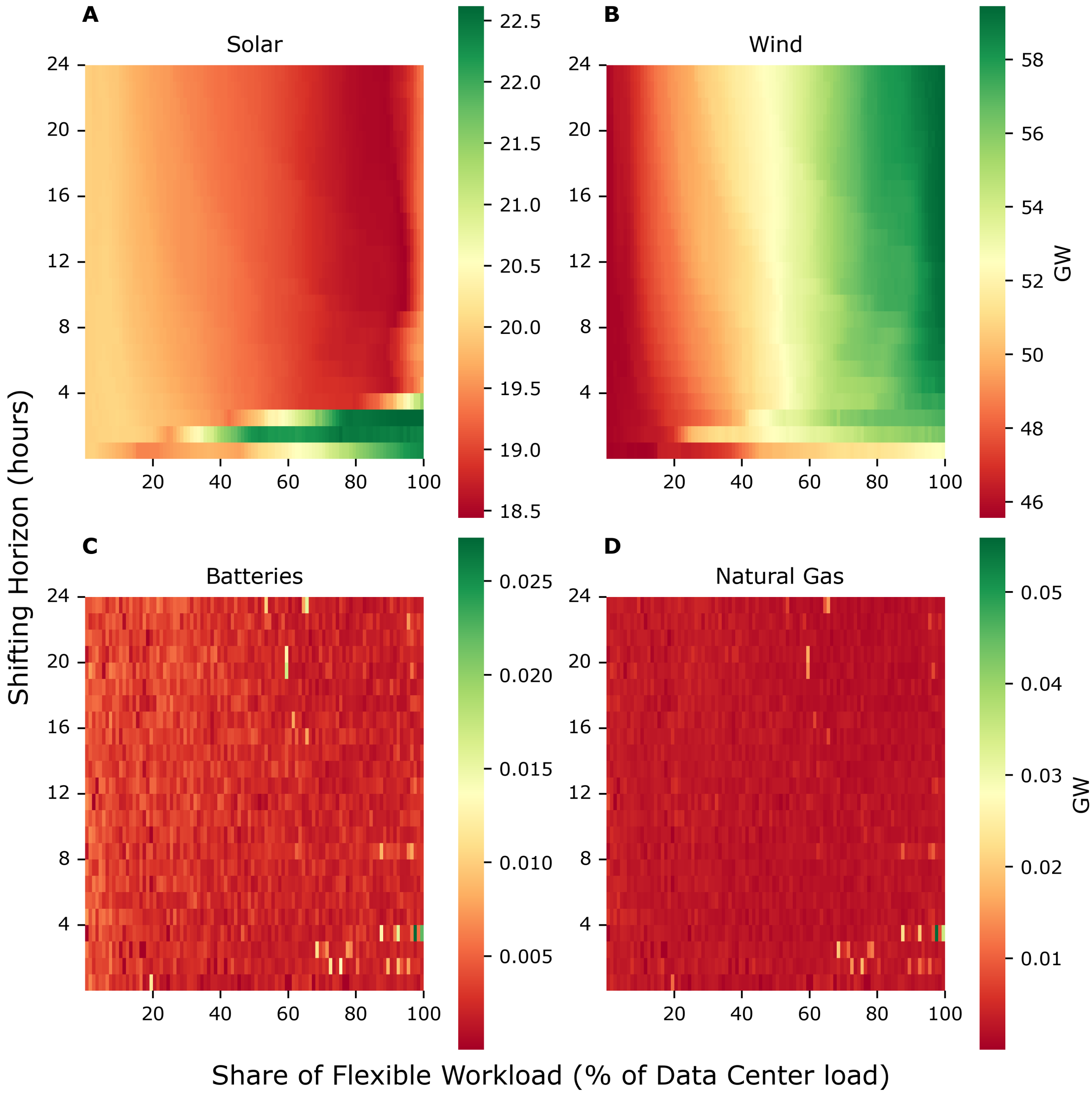

Figure S3: Capacity Investments per Technology in Texas. Panels show new capacity additions for solar **(A)**, wind **(B)**, batteries **(C)**), and natural gas **(D)** across combinations of data center shifting horizon (1 to 24 hours) and flexible workload share (1% to 100%). Note that color scales vary across subplots.

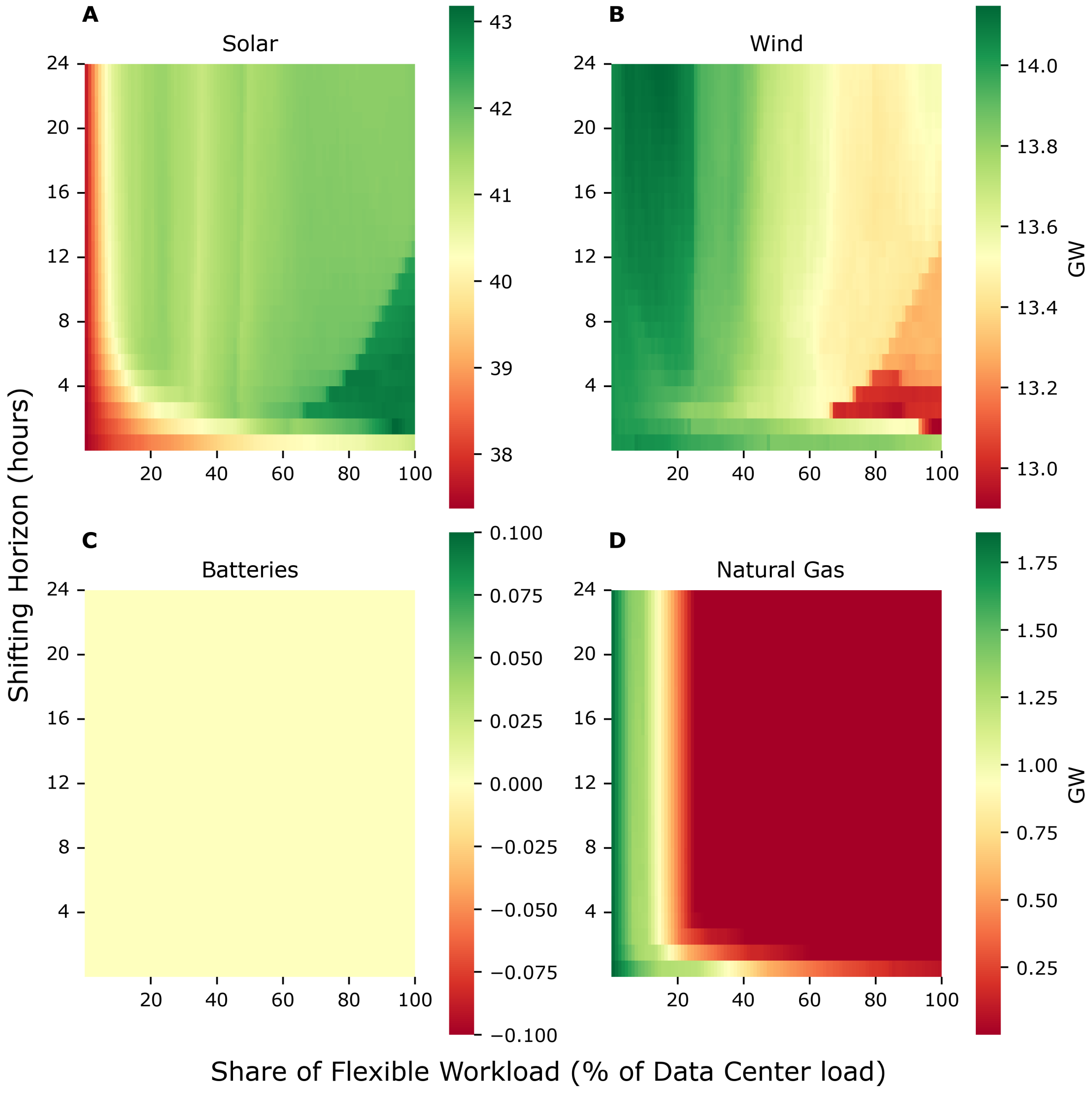

Figure S4: Capacity Investments per Technology in WECC. Panels show new capacity additions for solar **(A)**, wind **(B)**, batteries **(C)**), and natural gas **(D)** across combinations of data center shifting horizon (1 to 24 hours) and flexible workload share (1% to 100%). Note that color scales vary across subplots.

S4 WECC Data Center Shifting Operations

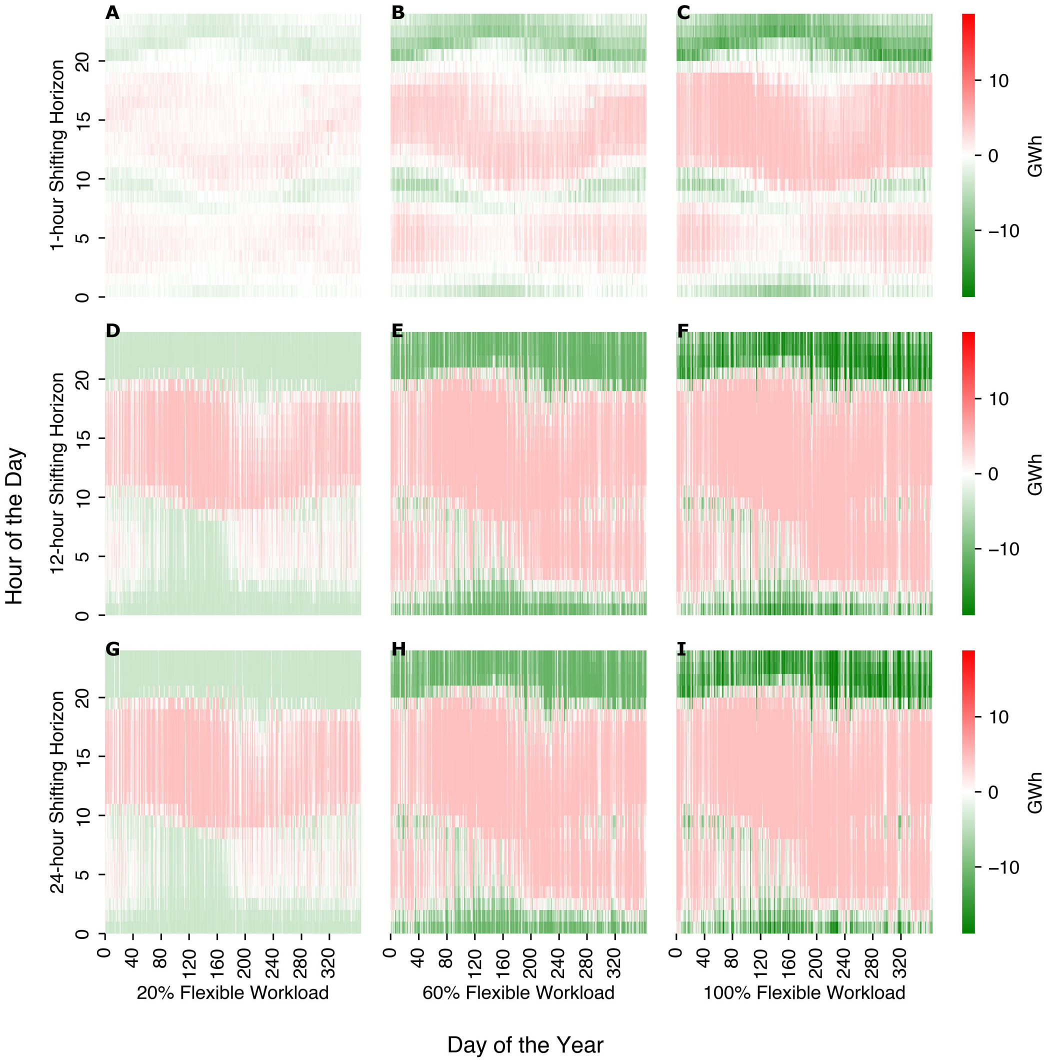

Figure S5: Hourly Data Center Load Change in WECC. Each panel shows the hourly change in data center electricity demand (GWh) relative to the baseline, across all 8760 hours of the year. The x-axis denotes the day of the year (1–365) and the y-axis the hour of the day (0–23). Red cells indicate hours in which load is added (workload shifted in). Green cells indicate hours in which load is reduced (workload shifted out). Columns correspond to the share of total data center load eligible for shifting: 20% (left), 60% (center), and 100% (right). Rows correspond to the maximum shifting horizon. The farthest ahead or behind a computational task may be rescheduled: 1 hour (top), 12 hours (middle), and 24 hours (bottom).

S5 Additional Capacity Information

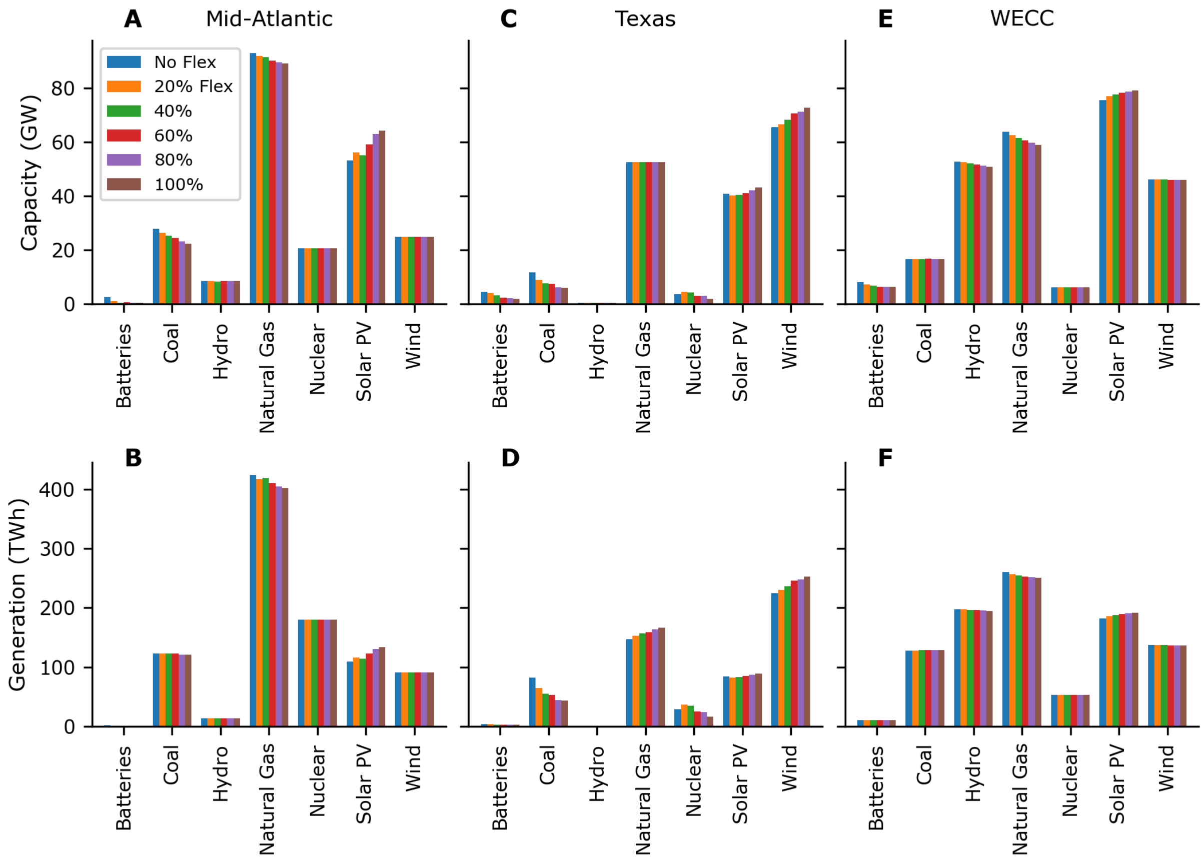

Figure S6: Capacity and Generation per Region with a 1-hour shifting horizon. Top row **(A, C, E)** shows total installed capacity by technology, net of new investments and retirements, for each region. Bottom row **(B, D, F)** presents corresponding total generation by technology. Results are shown for flexible workload shares ranging from 20% to 100% in 20% increments, alongside a baseline scenario without flexibility. All scenarios assume a 1-hour shifting horizon. No new capacity investments can be made in Coal, Nuclear, and Hydro. All technology types can be retired.

S6 Cost Differences per Component

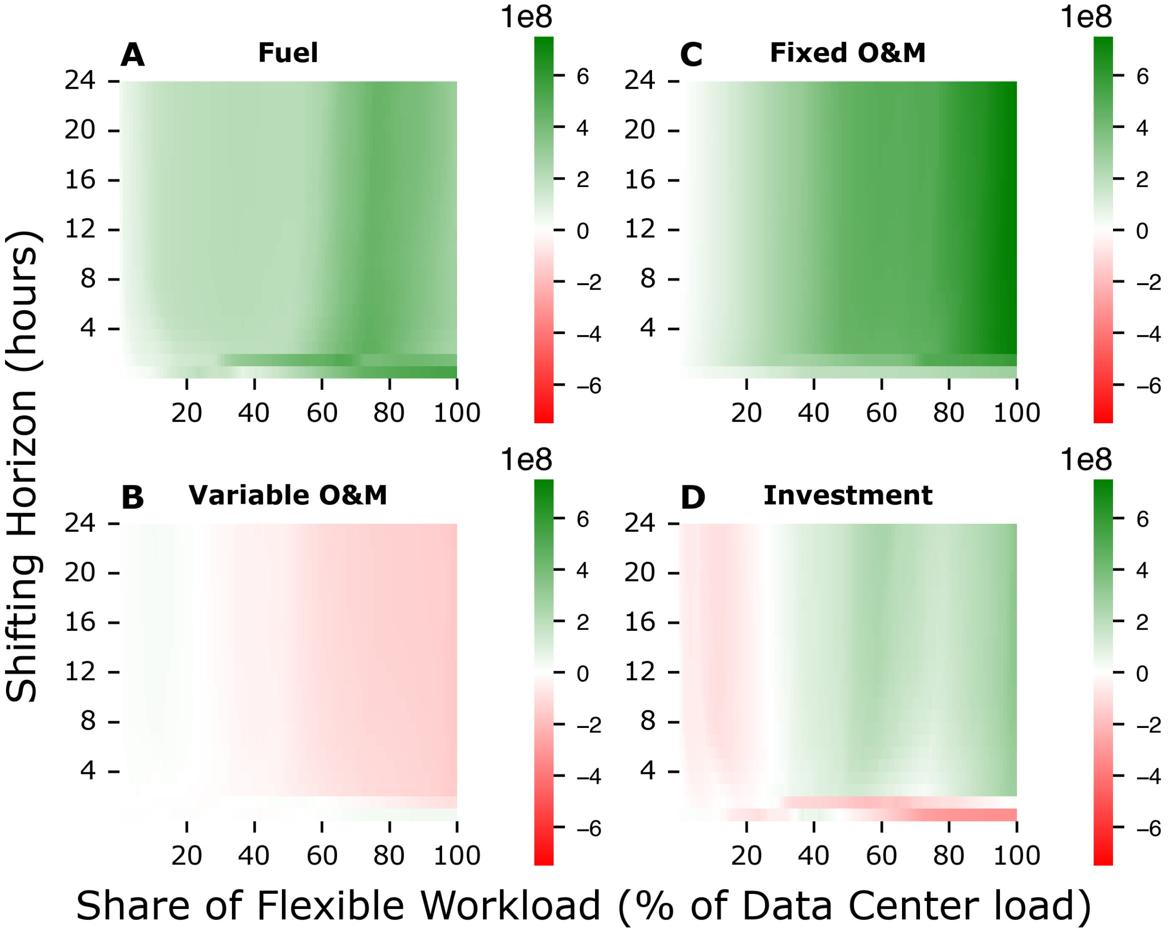

Figure S7: Cost Difference per Component for the Mid-Atlantic. Panels show the change in system costs between scenarios with and without data center flexibility for fuel **(A)**, fixed O&M **(B)**, variable O&M **(C)**, and generation investment **(D)**, across combinations of shifting horizon and flexible workload share. Green indicates a cost reduction with flexibility; red indicates an increase.

S7 Sensitivity Analysis Additional Results

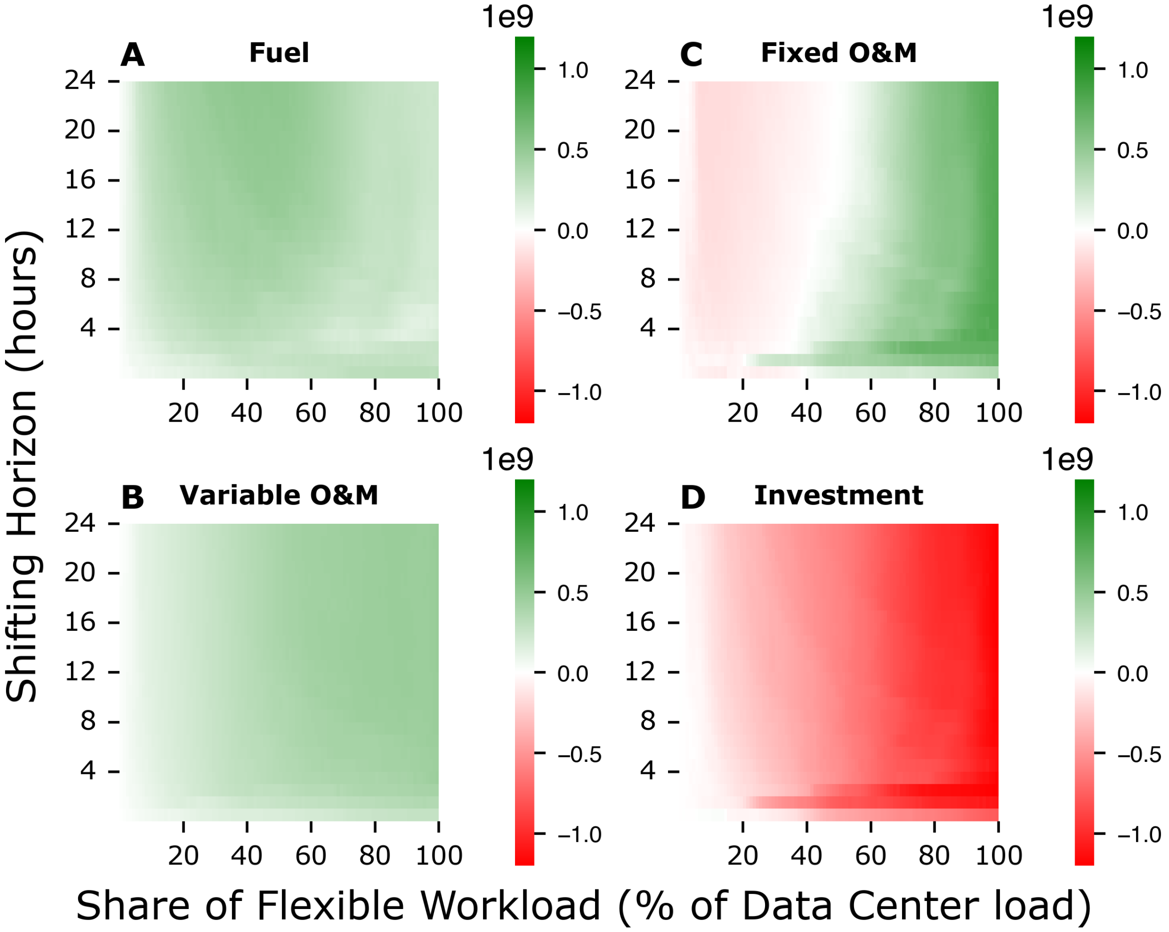

Figure S8: Cost Difference per Component for Texas. Panels show the change in system costs between scenarios with and without data center flexibility for fuel **(A)**, fixed O&M **(B)**, variable O&M **(C)**, and generation investment **(D)**, across combinations of shifting horizon and flexible workload share. Green indicates a cost reduction with flexibility; red indicates an increase.

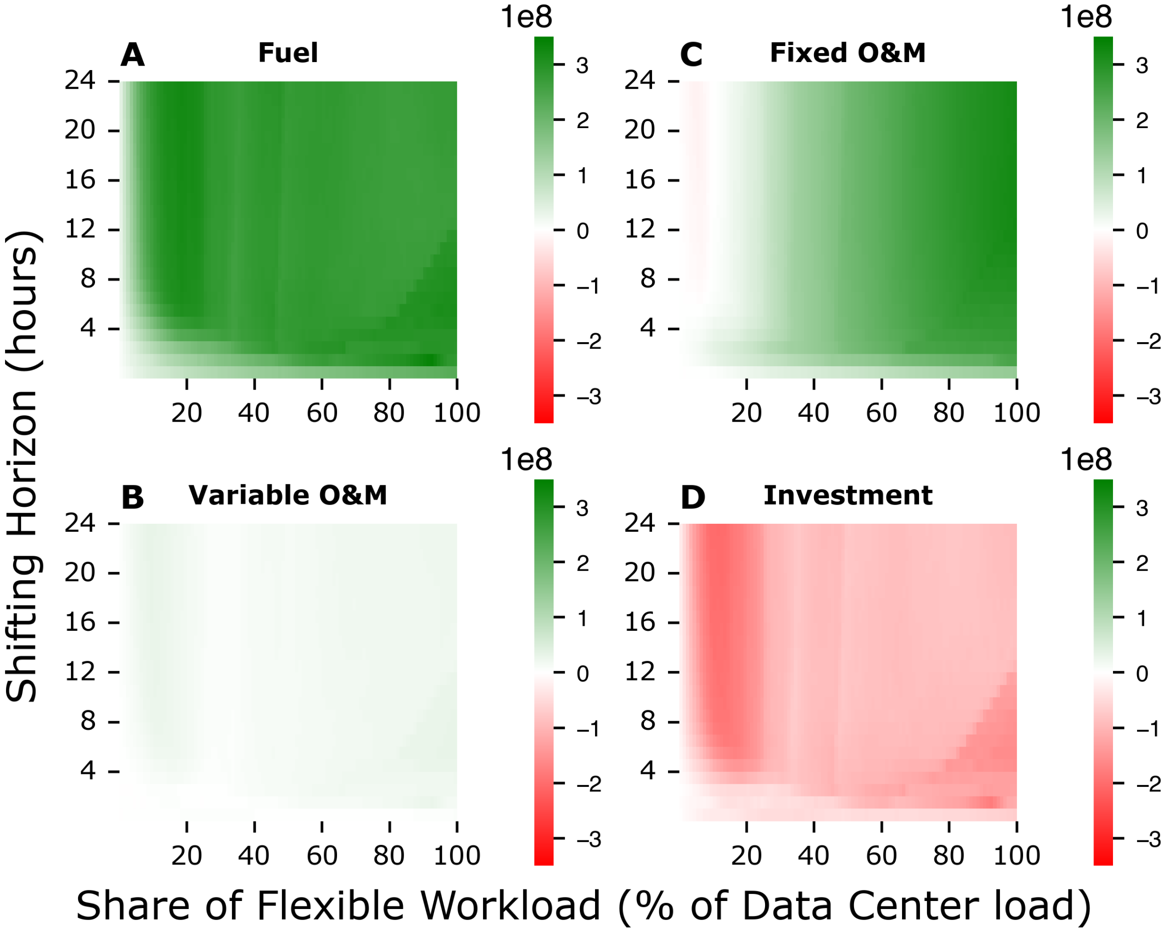

Figure S9: Cost Difference per Component for WECC. Panels show the change in system costs between scenarios with and without data center flexibility for fuel **(A)**, fixed O&M **(B)**, variable O&M **(C)**, and generation investment **(D)**, across combinations of shifting horizon and flexible workload share. Green indicates a cost reduction with flexibility; red indicates an increase.

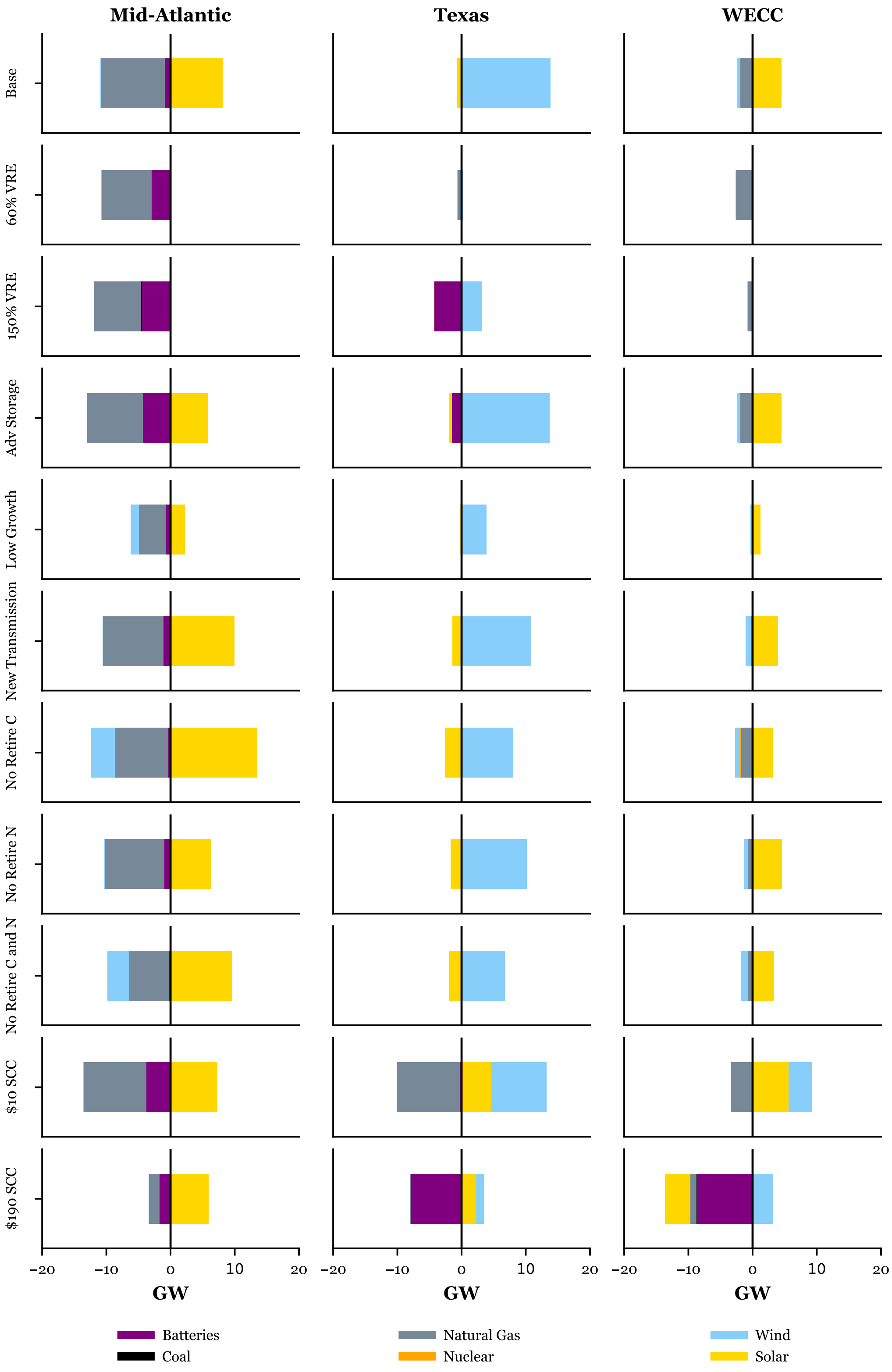

Figure S10: Difference in Investments in New Capacity between the Flexibility and No Flexibility Scenarios. Stacked bar charts in each panel represent the difference in investments in new capacity per technology type. A positive (negative) value indicates larger investments in the Flexibility (No Flexibility) scenario. Each row is a sensitivity analysis scenario, and each column is a region.

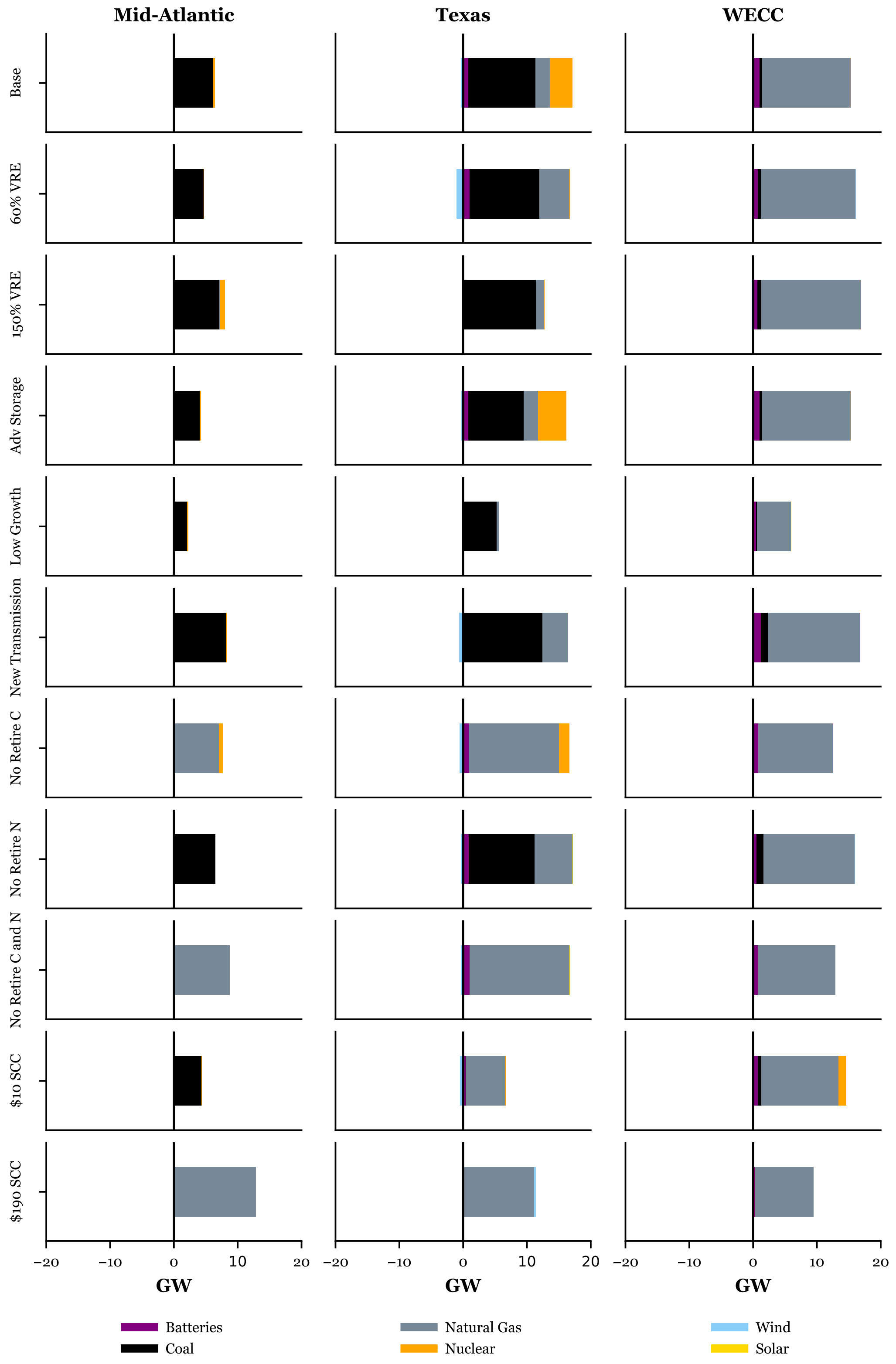

Figure S11: Difference in Retirement of Existing Capacity between the Flexibility and No Flexibility Scenarios. Stacked bar charts in each panel represent the difference in total capacity retirement per technology type. A positive (negative) value indicates larger retirements in the Flexibility (No Flexibility) scenario. Each row is a sensitivity analysis scenario, and each column is a region.

References

[1] EPRI, *Powering Data Centers: U.S. Energy System and Emissions Impacts of Growing Loads*, Tech. Rep. 3002031198 (2024), https://www.epri.com/research/products/000000003002031198.

[2] G. Schivley, Power Genome, https://github.com/PowerGenome/ (2023).

[3] T. Mai, *et al.*, Electrification Futures Study Load Profiles. *National Renewable Energy Laboratory* (2020), doi:10.7799/1593122.

[4] EPRI, *Powering Intelligence: Analyzing Artificial Intelligence and Data Center Energy Consumption*, Tech. Rep. 3002028905 (2024), https://www.epri.com/research/products/3002028905.

[5] NREL, NREL Annual Technology Basline 2022, https://atb.nrel.gov/electricity/2022/data (2022).

[6] EPA, *Documentation for EPA's Power Sector Modeling Platform v6 - Summer 2021 Reference Case*, Tech. rep., United States Environmental Protection Agency (2021), https://www.epa.gov/power-sector-modeling/documentation-epas-power-sector-modeling-platform-v6-summer-2021-reference.

[7] C. T. M. Clack, A. Choukulkar, B. Coté, S. A. McKee, *Dataset Overviews: Renewable Generation, Electric Demand, Transmission Line Ratings & Losses, and Climate Change*, Tech. rep., Vibrant Clean Energy, LLC (2020).
